# The POU2F1-ALDOA axis promotes the proliferation and chemoresistance of colon cancer cells by enhancing glycolysis and the pentose phosphate pathway activity

**DOI:** 10.1038/s41388-021-02148-y

**Published:** 2022-01-08

**Authors:** Jinguan Lin, Longzheng Xia, Linda Oyang, Jiaxin Liang, Shiming Tan, Nayiyuan Wu, Pin Yi, Qing Pan, Shan Rao, Yaqian Han, Yanyan Tang, Min Su, Xia Luo, Yiqing Yang, Xiaohui Chen, Lixia Yang, Yujuan Zhou, Qianjin Liao

**Affiliations:** 1 Hunan Key Laboratory of Cancer Metabolism, Hunan Cancer Hospital and the Affiliated Cancer Hospital of Xiangya School of Medicine, Central South University, Changsha, 410013 Hunan China; 2 University of South China, Hengyang, 421001 Hunan China; 3 Hunan Key Laboratory of Translational Radiation Oncology, 283 Tongzipo Road, Changsha, 410013 Hunan China

**Keywords:** Cancer metabolism, Chemotherapy

## Abstract

Cancer metabolic reprogramming enhances its malignant behaviors and drug resistance, which is regulated by POU domain transcription factors. This study explored the effect of POU domain class 2 transcription factor 1 (POU2F1) on metabolic reprogramming in colon cancer. The POU2F1 expression was analyzed in GEO dataset, TCGA cohorts and human colon cancer tissues by bioinformatics and immunohistochemistry. The effects of altered POU2F1 expression on proliferation, glucose metabolism and oxaliplatin sensitivity of colon cancer cells were tested. The impacts of POU2F1 on aldolase A (ALDOA) expression and malignant behaviors of colon cancer cells were examined. We found that up-regulated POU2F1 expression was associated with worse prognosis and oxaliplatin resistance in colon cancer. POU2F1 enhanced the proliferation, aerobic glycolysis and the pentose phosphate pathway (PPP) activity, but reduced oxidative stress and apoptosis in colon cancer cells, dependent on up-regulating ALDOA expression. Mechanistically, POU2F1 directly bound to the ALDOA promoter to enhance the ALDOA promoter activity in colon cancer cells. Moreover, activation of the POU2F1-ALDOA axis decreased the sensitivity to oxaliplatin in colon cancer cells. These data indicate that the POU2F1-ALDOA axis promotes the progression and oxaliplatin resistance by enhancing metabolic reprogramming in colon cancer. Our findings suggest that the POU2F1-ALDOA axis may be new therapeutic targets to overcome oxaliplatin resistance in colon cancer.

## Introduction

Colon cancer is the third most common cancer worldwide and its incidence is rapidly increasing in developed countries [1]. Although therapeutic strategies, such as surgical resection and chemotherapy, have prolonged the survival of many colon cancer patients, chemoresistance to therapeutic drugs has led to a poor prognosis [2]. Hence, discovering new therapeutic targets and understanding the mechanisms underlying the chemoresistance are critical for management of colon cancer patients.

Cellular metabolic reprogramming can promote aggressive proliferation of tumor cells [3–5]. The enhanced glycolysis produces intermediate metabolites for nucleic acid and fatty acid synthesis, thereby supporting the tumor growth [6]. However, the molecular mechanisms by which glycolysis regulates the proliferation of colon cancer cells have not been clarified. Previous studies have shown that three isoforms of aldolases (ALDOA, ALDOB, and ALDOC) are closely associated with the progression of several types of cancers, such as lung cancer and hepatocellular carcinomas [7, 8]. A recent study has revealed that up-regulated ALDOA expression promotes the proliferation, sphere formation, and radio-resistance of colorectal cancer cells [9]. However, it is still unclear how ALDOA expression is regulated and how ALDOA regulates glycolysis in colon cancer.

POU domain class 2 transcription factor 1 (POU2F1), also known as octamer transcription factor 1 (OCT1), is a member of the DNA-binding POU domain containing proteins and related to the embryonic stem cell master transcription factor, like OCT4 [10]. Functionally, POU2F1 can regulate carcinogenesis and cancer progression. Evidently, POU2F1 is up-regulated in human gastric, intestinal and colon stem cells [11] and positively correlated with tumor aggressiveness. POU2F1 deficiency can increase oxidative metabolism and elevate the levels of intracellular reactive oxygen species (ROS) and hypersensitivity to oxidative and genotoxic stress in colonic stem cells [12]. POU2F1 can also promote glycolytic metabolism and mitotic stability by promoting poised gene expression in colon cancer cells [13]. However, it is still unclear how POU2F1 regulates glycolysis and promotes the progression of colon cancer.

In this study, we employed the gain and loss of function strategies to determine the roles of POU2F1 and/or ALDOA in the glucose metabolism, proliferation and drug resistance of colon cancer in vitro and in vivo. Our data indicated that POU2F1 directly bound to the ALDOA promoter and enhanced its activity in colon cancer cells. The POU2F1-ALDOA axis was crucial for the metabolic reprogramming, glycolysis, growth and chemoresistance of colon cancer. Together, our findings may uncover a novel pathway to promote the proliferation, glycolysis and oxaliplatin resistance in colon cancer. Hence, targeting POU2F1 and/or ALDOA may be a promising strategy to overcome oxaliplatin resistance in colon cancer.

## Results

### High-POU2F1 expression is associated with a worse prognosis of colon cancer

**Fig. 1:**
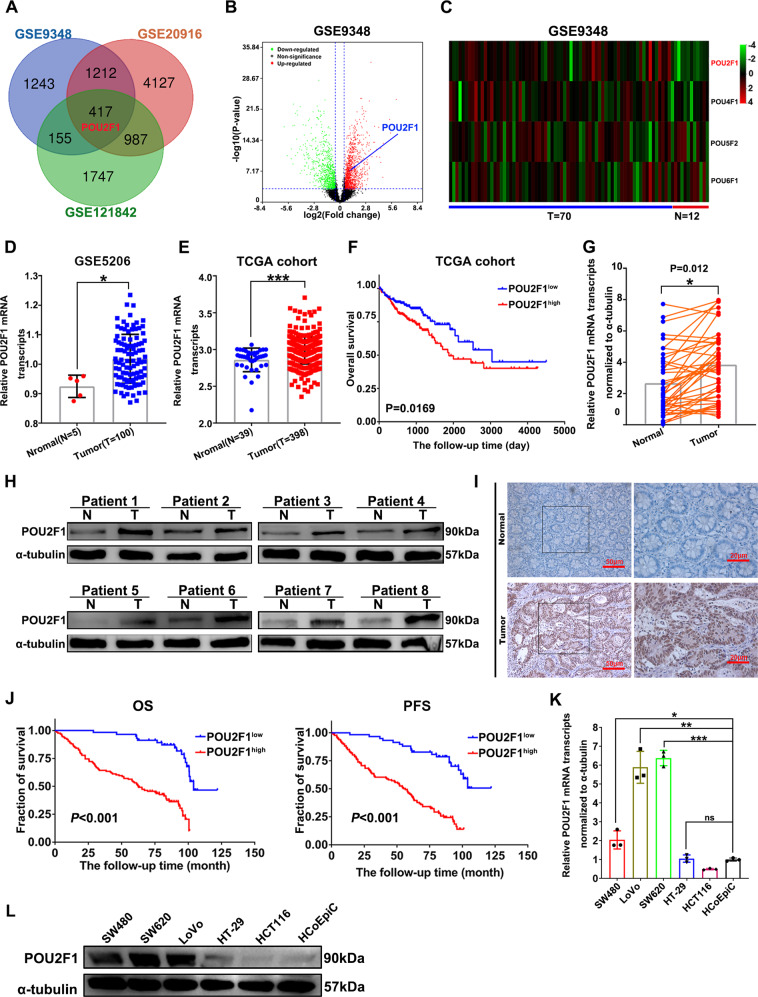
Up-regulated POU2F1 expression is associated with worse prognosis of colon cancer. **A** Venn diagrams identified 417 common DEG in three datasets (GSE9348, GSE20916, and GSE121842). **B** Volcano plot displayed the distribution of DEGs in 70 colon cancer tissues versus 12 non-tumor colon tissues in the GSE9348 dataset. The DEGs were determined, based on absolute fold change ≥ 1.5 and *p* value < 0.05. **C** Heatmap analysis of DEGs in the GSE9348 family. **D** Scatter plot exhibited the levels of POU2F1 mRNA transcripts in 100 colon cancer (Tumors) and 5 non-tumor colon tissues (Normal) in GSE5206 dataset. **E** Scatter plot displayed the levels of POU2F1 mRNA transcripts in 398 colon cancer (Tumors) and 39 non-tumor colon tissues (Normal) in TCGA. **F** Kaplan–Meier analysis of OS in colon cancer patients after stratification with the median value of POU2F1 mRNA transcripts in TCGA. The relative levels of POU2F1 mRNA transcripts (**G**, *n* = 42) and protein (**H**, *n* = 8) expression in clinical human colon carcinoma and corresponding adjacent tissues were analyzed by RT-qPCR and Western blotting, respectively. **I** IHC analysis of POU2F1 expression in colon cancer (magnification x100, scale bars 100 μm, magnification x200, scale bars 50 μm). **J** Kaplan–Meier analysis of PFS and OS in colon cancer patients after stratifying them by the defined POU2F1 IHC score. **K**, **L** Quantitative analysis of the relative levels of POU2F1 mRNA transcripts and protein to α-tubulin in five human colon cancer cell lines and non-tumor colonic epithelial HCoEpiC cells by RT-qPCR and Western blotting. Data are representative images or expressed as the mean ± SD of each group of samples analyzed in triplicate from three separate experiments. *p < 0.05, **p < 0.01, ***p < 0.001.

To understand the role of POU2F1 in colon cancer, we analyzed the expression of POU family members in the GEO datasets. As shown in Figs. 1A–D and S1A–D, POU2F1 expression was up-regulated in colon cancer tissues. The POU2F1 gene was the only differentially expressed gene (DEG) of the POU family in all three datasets, according to a fold change ≥ 1.5 and *p* < 0.05. Similarly, the levels of POU2F1 transcripts in 398 colorectal cancer tissues were significantly higher than that in 39 non-tumor colorectal tissues in TCGA (*p* < 0.01, Fig. 1E). Higher POU2F1 expression was associated significantly with a shorter overall survival (OS) of colorectal cancer patients in the population (*p* < 0.001, Fig. 1F). Moreover, high levels of POU2F1 expression were detected in colon cancer tissues (Fig. 1G–I). In addition, higher POU2F1 expression was significantly associated with higher TNM stage (*p* = 0.002) and metastases (*p* = 0.013), but not with age, gender and the differentiation status in 184 colon cancer patients (Table 1). Kaplan–Meier analysis displayed that high-POU2F1 expression was significantly associated with a shorter progression-free survival (PFS) and OS of colon cancer patients (*p* < 0.001, Fig. 1J). Univariate and multivariate analyses uncovered that higher POU2F1 expression and metastasis were independent risk factors for worse PFS and OS (*p* < 0.01, Table 2). High-POU2F1 expression was also obviously detected in colon cancer cell lines compared to human non-tumor colonic epithelial HCoEpiC cells (*p* < 0.05, Fig. 1K, L). Together, these results indicated that up-regulated POU2F1 expression was associated with worse prognosis of colon cancer, suggesting that POU2F1 may act as an oncogenic factor of colon cancer.

**Table 1:** Association of POU2F1 expression and clinicopathological characteristics of colon cancer patients.

Variable		POU2F1		*p* value
	*N*	High	Low	
Age				
<60	74	47	27	0.234
≥60	110	79	31	
Gender				
Male	114	79	35	0.760
Female	70	47	23	
Histology grading				
Well differentiation	29	21	8	0.748
Moderate differentiation	125	86	39	
Poor differentiation	30	19	11	
TNM stages				
I	47	34	13	0.002
II	72	40	32	
III	40	28	12	
IV	25	24	1	
Metastasis				
Yes	65	52	13	0.013
No	119	74	45

**Table 2:** Univariate and multivariate analyses of factors for the PFS and OS of colon cancer patients.

Variables	Progression-free survival						Overall survival					
	Univariate			Multivariate			Univariate			Multivariate		
	HR	95% CI	*p*	HR	95% CI	*p*	HR	95% CI	*p*	HR	95% CI	*p*
Age(≥60/<60 years)	1.374	0.939–2.008	0.102	1.199	0.814–1.767	0.359	1.173	0.778–1.768	0.447	0.928	0.607–1.419	0.730
Gender (Female/Male)	0.957	0.656–1.396	0.820	1.060	0.722–1.558	0.766	0.952	0.635–1.428	0.812	0.997	0.659–1.508	0.988
Histology grading (Well/Moderate/Poorly and Unknown)	1.204	0.813–1.784	0.354	1.262	0.900–1.771	0.177	1.086	0.766–1.540	0.644	1.232	0.853–1.779	0.266
Stage(I/II/III/IV)	1.895	1.547–2.323	0.000	1.117	0.764–1.635	0.576	2.165	1.743–2.689	0.000	1.145	0.759–1.727	0.519
Metastasis(Yes/No)	3.864	2.647–5.639	0.000	3.694	1.680–8.119	0.001	5.086	3.378–7.660	0.000	4.418	1.904–10.250	0.001
POU2F1 (High/Low)	4.937	2.920–8.348	0.000	5.940	3.448–10.233	0.000	5.625	3.139–10.081	0.000	6.546	3.570–12.005	0.000

*HR* hazard ratio, *CI* confidence interval.

### POU2F1 enhances malignant behaviors of colon cancer cells

**Fig. 2:**
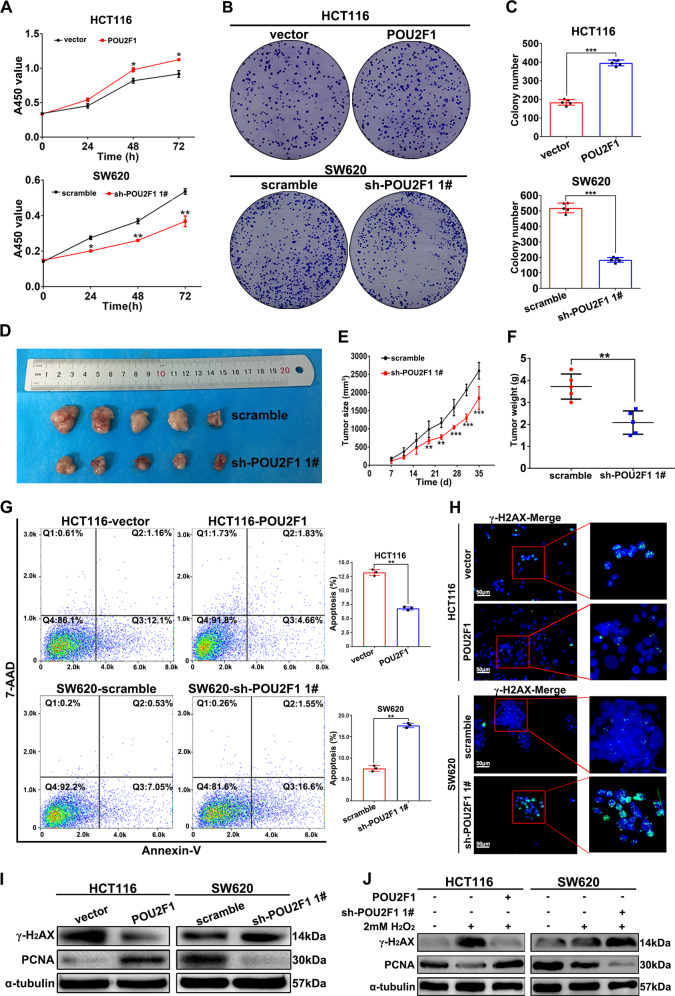
POU2F1 promotes the growth and inhibits the apoptosis of colon cancer cells. **A**–**C** POU2F1 enhanced the proliferation and clonogenicity of SW620 cells and HCT116 cells, determined by CCK8 assays and colony formation assays, respectively. **D**–**F** POU2F1 silencing inhibited the growth of implanted colon cancer in mice. BALB/c nude mice were injected subcutaneously with 5 × 10^6^ cells indicated. The dynamic growth of tumors was measured longitudinally (*n* = 5 per group). At the end of observation, their tumors were dissected, photoimaged, and weighed. **G** Flow cytometry analysis of apoptotic cells. **H** Immunofluorescent analysis of γ-H_2_AX expression in the indicated cells (magnification x200, scale bars 50 μm). **I** Western blot analysis of the relative levels of γ-H_2_AX and PCNA expression in the indicated cells. **J** Western blot analysis of the relative levels of γ-H_2_AX and PCNA expression in the indicated cells after 2 mM H_2_O_2_ treatment. Data are representative images or expressed as the mean ± SD of each group of samples analyzed in triplicate from three separate experiments. *p < 0.05, **p < 0.01, ***p < 0.001.

Next, we tested the impact of altered POU2F1 expression on malignant behaviors of colon cancer cells. We generated POU2F1 stably over-expressing/silencing cell lines (Fig. S2A, B). POU2F1 over-expression significantly promoted cell proliferation, colony formation and implanted tumor growth, while POU2F1 silencing had remarkably opposite effects on colon cancer cells (*p* < 0.05, Figs. 2A–F and S2C). POU2F1 over-expression decreased the frequency of apoptotic HCT116 cells while POU2F1 silencing increased the percentages of apoptotic SW620 cells (*p* < 0.01, Fig. 2G). Aberrant DNA damages and impaired repair are related to apoptosis and inhibit the proliferation of cells [14–17]. We found that POU2F1 over-expression up-regulated the expression of proliferating cell nuclear antigen (PCNA), but decreased phosphorylated H_2_A histone family member X (γ-H_2_AX) in HCT116 cells; conversely, POU2F1 silencing down-regulated PCNA, but increased γ-H_2_AX expression in SW620 cells (Figs. 2H, I and S2D). We further texted whether the change in POU2F1 expression could modulate the H_2_O_2_-induced γ-H_2_AX and PCNA expression in colon cancer cells. As shown in Fig. 2J, H_2_O_2_ exposure increased γ-H_2_AX expression, but decreased PCNA expression in both HCT116 and SW620 cells. The effects of H_2_O_2_ on γ-H_2_AX and PCNA expression were dramatically mitigated by POU2F1 over-expression, but enhanced by POU2F1 silencing. As a result, H_2_O_2_ exposure did increase the frequency of apoptotic POU2F1-silenced SW620 cells, but not POU2F1 over-expressing HCT116 cells (Fig. S2E). Moreover, enforced POU2F1 expression rescued the proliferation and colony formation of POU2F1 silencing SW620 cells (*p* < 0.05, Fig. S2F–H). These data demonstrated that POU2F1 acted as an oncogenic factor to promote the malignant behaviors of colon cancer cells.

### POU2F1 enhances glycolysis and the pentose phosphate pathway (PPP) activity in colon cancer cells

**Fig. 3:**
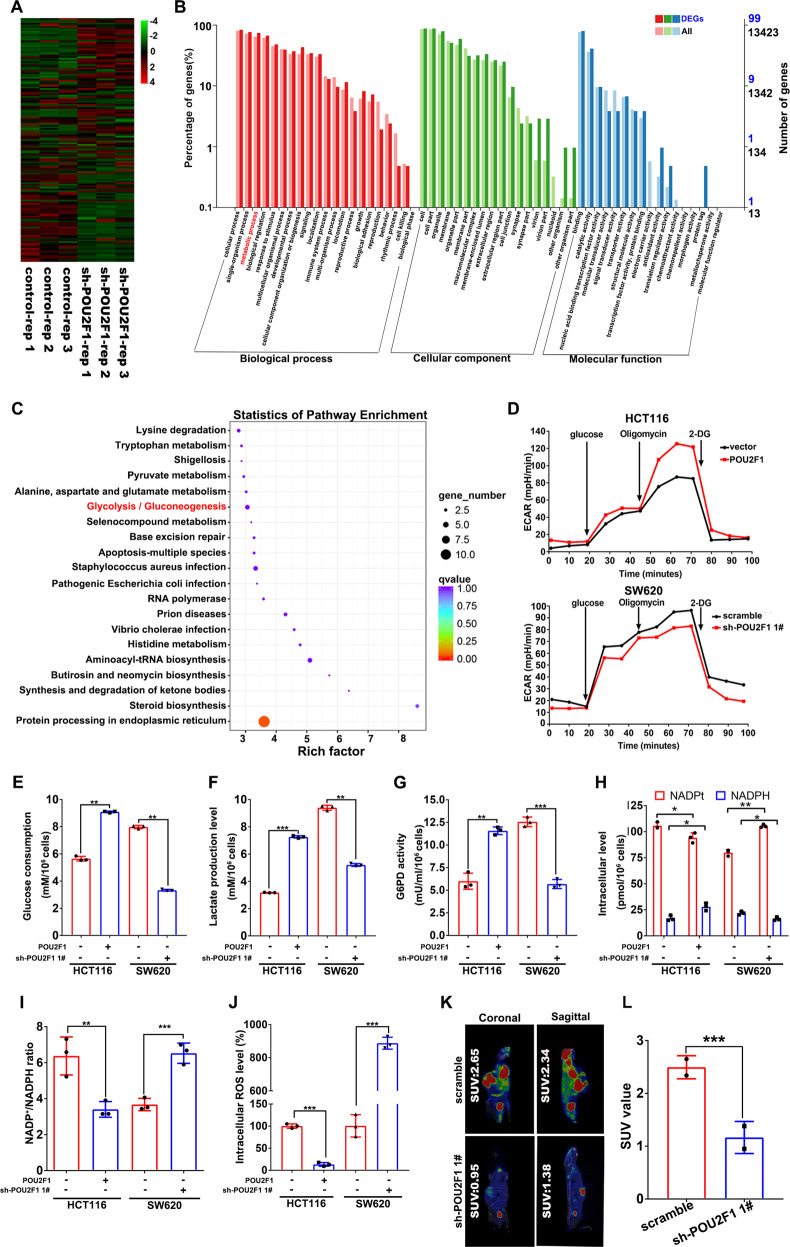
POU2F1 enhances glycolysis and PPP activity in colon cancer cells. **A** Heatmap analysis of the 141 DEGs between POU2F1 silencing and control SW620 cells. **B** The GO analysis of 141 DEGs. **C** KEGG analysis of 141 DEGs. **D** Seahorse analysis of glycolytic activity in the indicated cells following treatment with glucose (10 mM), oligomycin (1.0 µM), 2-Deoxy-d-glucose (2-DG, 50 mM). **E**, **F** The levels of glucose consumption and lactate production in the indicated cells. **G** The levels of G6PD activity in the indicated cells. **H**, **I** The levels of intracellular NADP^+^ and NADPH and the ratios of NADP^+^/NADPH in the indicated cells. **J** Flow cytometry analysis of intracellular ROS levels. The levels of intracellular ROS in the control HCT116 cells were designated as 100%. **K**, **L** Representative images of ^18^F-FDG PET/CT scanning of the mice with SW620-sh-POU2F1 1# or control SW620-scramble tumors (*n* = 5 per group). The SUV values in individual mice were calculated. Data are representative images or expressed as the mean ± SD of each group of samples analyzed in triplicate from three separate experiments. *p < 0.05, **p < 0.01, ***p < 0.001.

To further understand the biological significance and mechanisms underlying the action of POU2F1 in colon cancer, we investigated how POU2F1 silencing could alter mRNA transcription in SW620 cells by RNA-sequence analysis. We found 141 DEGs with 83 up-regulated and 58 down-regulated in SW620-sh-POU2F1 cells (with a fold change of >1.5 and *p* value of <0.01) (Fig. 3A). Gene ontology (GO) analysis indicated that the DEGs were mainly enriched in cellular process, metabolic process, biological regulation, and others (Fig. 3B). The Kyoto Encyclopedia of Genes and Genomes (KEGG) analysis exhibited that these DEGs were enriched in a number of metabolic pathways, such as glycolysis and others (Fig. 3C). Additionally, the GSEA analysis of GSE121842 displayed that gene sets related to glycolysis and PPP were enriched in the high-POU2F1 expressing colon cancer cases (Fig. S3A). It suggests that POU2F1 may increase glycolysis and PPP activity to enhance the malignant behaviors of colon cancer cells. Consistently, seahorse analysis revealed that POU2F1 over-expression enhanced the glycolysis, whereas POU2F1 silencing reduced it in colon cancer cells (Fig. 3D). POU2F1 over-expression significantly increased glucose consumption, lactate production levels and glucose-6-phosphate-dehydrogenase (G6PD) activity, whereas POU2F1 silencing had opposite effects on colon cancer cells (*p* < 0.01, Fig. 3E–G). As shown in Fig. S3B–D, enforced POU2F1 expression significantly rescued glucose consumption, lactate production and G6PD activity in POU2F1 silencing cells (*p* < 0.05). In addition, we detected the levels of intracellular nicotinamide adenine dinucleotide phosphate (NADPH) and ROS in colon cancer cells. Compared with the controls, POU2F1 over-expression decreased NADP^+^/NADPH ratios and intracellular ROS levels while POU2F1 silencing increased them in colon cancer cells (*p* < 0.05, Fig. 3H–J). Additionally, enforced POU2F1 expression significantly increased the levels of NADP^+^ and NADPH as well as NADP^+^/NADPH ratios in POU2F1 silencing SW620 cells (*p* < 0.05, Fig. S3E, F). POU2F1 over-expression significantly mitigated the H_2_O_2_-increased intracellular ROS levels while POU2F1 silencing had opposite effects on colon cancer cells (*p* < 0.05, Fig. S3G). The ^18^F-FDG PET/CT of tumor-bearing mice also exhibited that the maximum standard uptake values (SUV max) in the mice bearing POU2F1 silencing tumors were significantly less than that in the mice with control tumors (*p* < 0.001, Fig. 3K). These results clearly demonstrated that POU2F1 enhanced glycolysis and PPP activity in colon cancer cells.

### POU2F1 regulates the expression of ALDOA in colon cancer cells

**Fig. 4:**
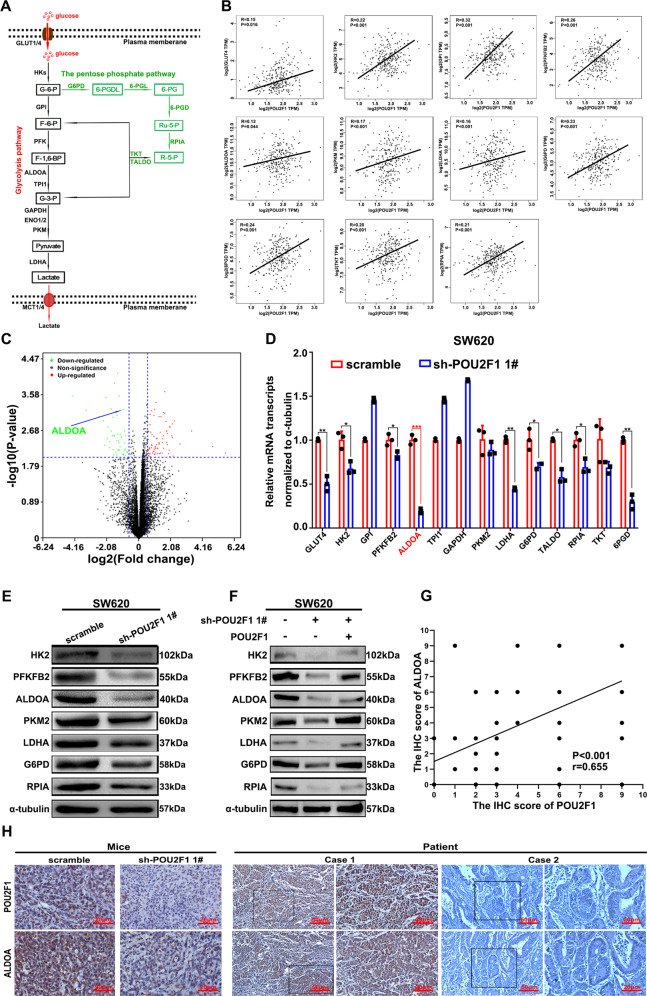
POU2F1 enhances the expression of ALDOA in colon cancer cells. **A** A schematic diagram illustrates the regulation of glycolysis and the pentose phosphate pathway (PPP). **B** The correlation between the relative levels of POU2F1 and GLUT4, HK2, GPI, PFKFB2, ALDOA, PKM, LDHA, G6PD, 6PGD, TKT and RPIA mRNA transcripts in 275 colon cancer tissues of TCGA database. **C** Volcano displayed the distribution of 141 DEGs between POU2F1 silencing and control SW620 cells. The DEGs were identified, based on a fold change ≥ 1.5 and *p* value < 0.05. **D**–**F** RT-qPCR and Western blot analyses of the relative levels of glycolysis-related *HK2, PFKFB2, ALDOA, PKM2, LDHA, G6PD* and *RPIA* gene expression in the indicated cells. **G** The correlation between the relative levels of POU2F1 and ALDOA, determined by IHC in 184 colon cancer tissues. **H** IHC analyses of POU2F1 and ALDOA expression in xenograft tumors and colon cancer specimens (magnification x200, scale bars 50 μm, magnification x400, scale bars 20 μm). Data are representative images or expressed as the mean ± SD of different groups of cell samples analyzed in triplicate from three separate experiments. ns no significance, *p < 0.05, **p < 0.01, ***p < 0.001.

To understand the mechanisms by which POU2F1 enhanced glycolysis and the PPP activity, we analyzed the potential correlation between POU2F1 and glycolysis/PPP-related genes in GEPIA website (http://gepia.cancer-pku.cn/). We found that POU2F1 transcripts were positively correlated with hexokinase 2 (HK2), 6-phosphofructo-2-kinase/fructose-2,6-biphosphatase 2 (PFKFB2), ALDOA, pyruvate kinase M1/2 (PKM), lactate dehydrogenase A (LDHA), G6PD, and ribose 5-phosphate isomerase A (RPIA) levels in the colon cancer tissues (Fig. 4A, B). Interestingly, the levels of ALDOA mRNA transcripts decreased in POU2F1 silencing SW620 cells (Fig. 4C). Similarly, qRT-PCR and Western blot assays exhibited that the expression levels of glycolysis/PPP-related genes significantly decreased (*p* < 0.05, Fig. 4D, E), which were rescued by enforced POU2F1 expression in POU2F1 silencing SW620 cells (Fig. 4F). In contrast, POU2F1 over-expression increased their expression in HCT116 cells (Fig. S4A, B). POU2F1 silencing also decreased HK2, G6PD and LDHA expression in xenograft tumors (Fig. S4C). Moreover, the levels of POU2F1 expression were correlated with ALDOA expression in 184 colon cancer tissues (*R* = 0.655, *p* < 0.001, Fig. 4G, H) and in colon cancer cell lines (Fig. S5A, B). The paralleled expression of POU2F1 and ALDOA suggests that POU2F1 may induce ALDOA expression to regulate the glycolysis and PPP activity in colon cancer.

### ALDOA enhances the proliferation, glycolysis, and PPP activity of colon cancer cells

To explore the role of ALDOA in colon cancer, we analyzed the expression and clinical significance of ALDOA in colon cancer. Firstly, the levels of ALDOA expression in 53 colorectal cancer tissues were significantly higher than that in 28 non-tumor colorectal tissues in GSE6988 dataset (*p* < 0.05, Fig. S5C) and higher ALDOA expression was associated significantly with a shorter OS of 177 colon cancer patients in GSE17538 dataset (*p* = 0.008, Fig. S5D). Similarly, higher ALDOA expression was significantly associated with shorter OS and PFS of 184 colon cancer patients (*p* < 0.0001 for both, Fig. S5E). Higher levels of ADLOA expression were detected in colon cancer tissues, compared with that in the non-tumor controls (Fig. S5F) and were significantly associated with clinical stages (*p* = 0.018) and metastasis (*p* = 0.018), but not with age, gender and the differentiation degree in colon cancer patients in this population (Table 3). Hence, ALDOA may act as an oncogenic factor of colon cancer.

**Table 3:** Association of ALDOA protein expression with clinicopathological characteristics of colon cancer patients.

Variable	*N*	ALDOA		*p* value
		High	Low	
Age				
<60	73	48	25	0.701
≥60	111	76	35	
Gender				
Male	114	77	37	0.955
Female	70	47	23	
Histology grading				
Well differentiation	29	21	8	0.820
Moderate differentiation	125	83	42	
Poor differentiation	30	20	10	
TNM stages				
I	47	33	14	0.018
II	72	40	32	
III	40	29	11	
IV	25	22	3	
Metastasis				
Yes	65	51	14	0.018
No	119	73	46	

To further clarify the role of ALDOA in the progression of colon cancer, we constructed stably ALDOA over-expressing and silencing cells, respectively (Fig. S5G). We found that ALDOA over-expression enhanced the proliferation and clonogenicity of colon cancer cells while ALDOA silencing had opposite effects (*p* < 0.05, Fig. S5H, I). A similar pattern of the levels of glucose consumption, lactate production, G6PD activity and intracellular NADPH as well as NADP^+^/NADPH ratios was observed in the different groups of cells (*p* < 0.05, Fig. S6A–E). ALDOA over-expression significantly decreased the levels of intracellular ROS and γ-H_2_AX expression, and increased the PCNA expression, while ALDOA silencing had opposite effects (*p* < 0.01, Fig. S6F, G). Together, these findings indicated that ALDOA enhanced the proliferation, glycolysis and PPP activity in colon cancer cells.

### POU2F1 enhances the ALDOA promoter activity by directly binding to the ALDOA promoter region

**Fig. 5:**
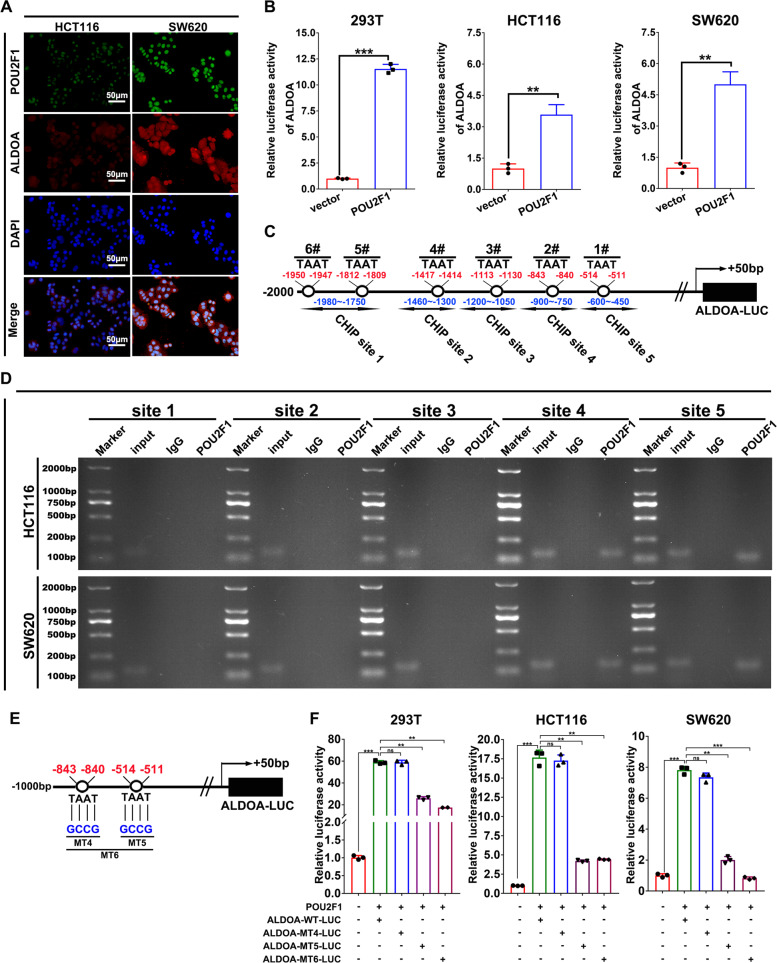
POU2F1 enhances the ALDOA promoter activity by directly binding to the ALDOA promoter. **A** Immunofluorescent analysis exhibited POU2F1 and ALDOA co-expression in SW620 and HCT116 cells (magnification x200, scale bars 50 μm). **B** Luciferase reporter assays indicated that POU2F1 enhanced the ALDOA promoter-controlled luciferase expression in the indicated cells. **C** Schematics of the POU2F1 putative binding sites in the 5′ upstream regions of the ALDOA promoter (−2000 bp ~ +50 bp). **D** Chromatin immunoprecipitation assay indicated that POU2F1 bound to the ALDOA promoter at sites 4, and 5. **E** Schematics of mutation strategies in the ALDOA promoter (−1000 bp ~ +50 bp). **F** Luciferase reporter assays exhibited that POU2F1 bound to the ALDOA promoter at site 5 to induce its expression. Data are representative images or expressed as the mean ± SD of each group of samples analyzed in triplicate from three separate experiments. **p < 0.01, ***p < 0.001.

Clearly, POU2F1 was paralleled with ALDOA expression in colon cancer cells and both of them acted as oncogenic factors to enhance the malignant behaviors of colon cancer. Next, we tested the hypothesis that POU2F1 could directly induce ALDOA expression in colon cancer cells. To address the hypothesis, we performed immunofluorescence assays to detect co-expression of POU2F1 and ALDOA in HCT116 and SW620 cells. Both POU2F1 and ALDOA expression was higher in SW620 cells than that in HCT116 cells (Figs. 5A and S7A). We transfected with the plasmid for POU2F1 expression, together with the luciferase reporter plasmid (pGL3-ALDOA-Luc) containing the sequence from +50 bp to −2000 bp of the human ALDOA promoter. We found that POU2F1 over-expression significantly increased the ALDOA promoter-regulated luciferase activity in the tested cell lines (*p* < 0.01, Fig. 5B). We also found that the core DNA-binding sequence (TAAT) of POU2F1 was located from −400 to −1980 bp in the ALDOA promoter region [18] (Fig. 5C). Subsequently, ChIP assays evidenced that anti-POU2F1 precipitated the genomic DNA containing the −900 to −450 bp region (sites 4 and 5) of the ALDOA promoter in the tested cell lines (Fig. 5D). Similar results were observed in POU2F1 over-expressing or silencing cells (Fig. S7B). To validate which the site in the ALDOA promoter was responsible for POU2F1 binding, we co-transfected the plasmid ALDOA-WT-Luc containing the −900 to −450 bp of the ALDOA promoter or ALDOA-MT4-Luc, ALDOA-MT5-Luc, ALDOA-MT6-Luc with the plasmid for POU2F1 expression and Renilla luciferase reporter into 293T, HCT116 and SW620 cells (Fig. 5E). While transfection with ALDOA-WT5-Luc significantly enhanced the luciferase promoter activity, transfection with the ALDOA-MT5-Luc, or ALDOA-MT6-Luc, but not ALDOA-MT4-LUC, significantly reduced the luciferase promoter activity in the tested cells (*p* < 0.01, Fig. 5F). Collectively, these results strongly indicated that POU2F1 directly bound to the site 5 of the ALDOA promoter to induce ALDOA transcription in these cell lines.

### ALDOA is essential for POU2F1 to enhance aerobic glycolysis and PPP activity in colon cancer cells

**Fig. 6:**
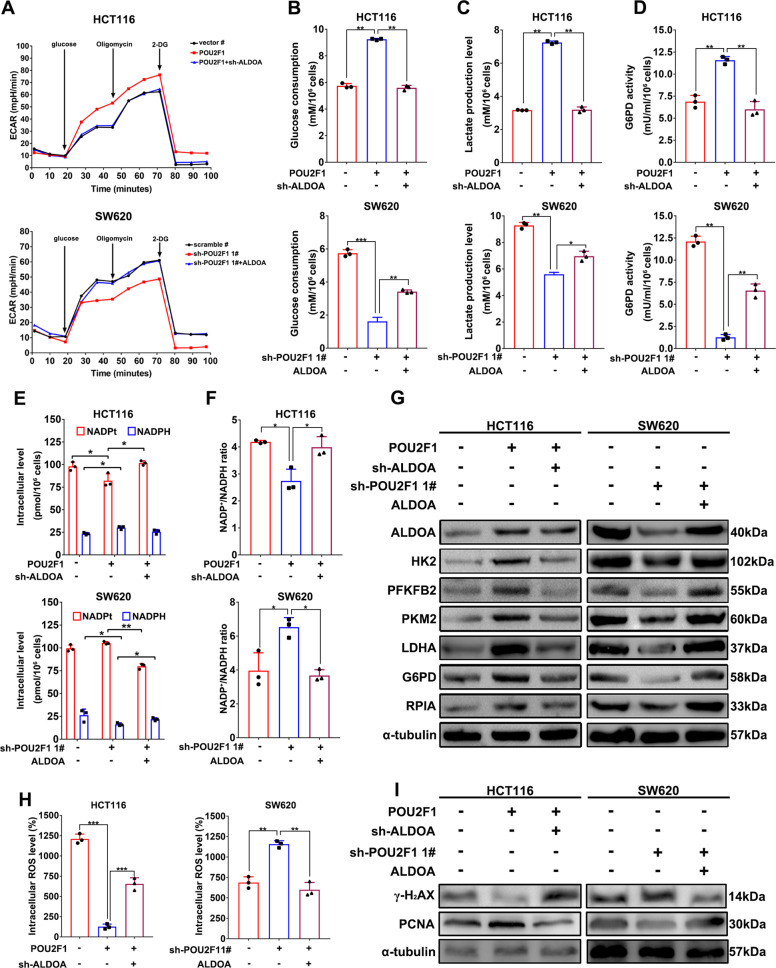
ALDOA is essential for the effect of POU2F1 on aerobic glycolysis and PPP in colon cancer cells. **A** Seahorse analysis of glycolysis in the indicated cells exhibited that POU2F1 enhanced glycolysis, dependent on up-regulating ALDOA expression. **B**–**F** The levels of glucose consumption and lactate production, G6PD activity, intracellular NADPH and the ratios of NADP^+^/NADPH in indicated cells. **G** Western blot analyses of the relative levels of glycolysis- and PPP-related protein expression in the indicated cells. **H** The levels of intracellular ROS in the indicated cells. **I** Western blot analyses of the relative levels of γ-H_2_AX and PCNA expression in the indicated cells. Data are representative images or expressed as the mean ± SD of each group of samples analyzed in triplicate from three separate experiments. *p < 0.05, **p < 0.01, ***p < 0.001.

We further tested the function of ALDOA in the POU2F1-enhanced glycolysis and PPP activity in colon cancer cells. Accordingly, we induced ALDOA over-expression in POU2F1 silencing cells or ALDOA silencing in POU2F1 over-expressing cells, respectively (Fig. S8A). Seahorse assays exhibited that ALDOA silencing abrogated the POU2F1 over-expression-increased extracellular acidification rate (ECAR) (Fig. 6A). A similar pattern was observed in measurement of extracellular glucose consumption, lactate production and G6PD activity (*p* < 0.05, Fig. 6B–D). Furthermore, ALDOA silencing also rescued the POU2F1 over-expression-decreased NADP^+^/NADPH ratios (*p* < 0.05, Fig. 6E, F). Western blot assays revealed that POU2F1 over-expression enhanced HK2, PRKFB2, PKM2, LDHA, G6PD and RPIA expression, which were mitigated or abrogated by ALDOA silencing (Fig. 6G). These data indicated that POU2F1 enhanced the glycolysis and PPP activity, dependent on its up-regulating ALDOA expression in colon cancer cells. Consequently, ALDOA silencing significantly rescued the POU2F1 over-expression-decreased intracellular ROS, γ-H_2_AX expression and mitigated the POU2F1 over-expression-increased PCNA expression while ALDOA over-expression significantly had opposite effects on the tested cells (*p* < 0.05, Figs. 6H, I and S8B, C). Similarly, ALDOA silencing also significantly increased the frequency of apoptotic POU2F1 over-expressing HCT116 cells, and decreased their proliferation and colony formation, while ALDOA over-expression dramatically had opposite effects on POU2F1 silencing SW620 cells (*p* < 0.05, Fig. S9A–C). Together, these data indicated that POU2F1 enhanced the survival, proliferation, and clonogenicity of colon cancer cells by up-regulating ALDOA expression, enhancing the glycolysis and PPP activity, but attenuating the DNA damage-induced apoptosis.

### The POU2F1-ALDOA axis contributes to the oxaliplatin resistance by enhancing glycolysis and PPP activity in colon cancer

**Fig. 7:**
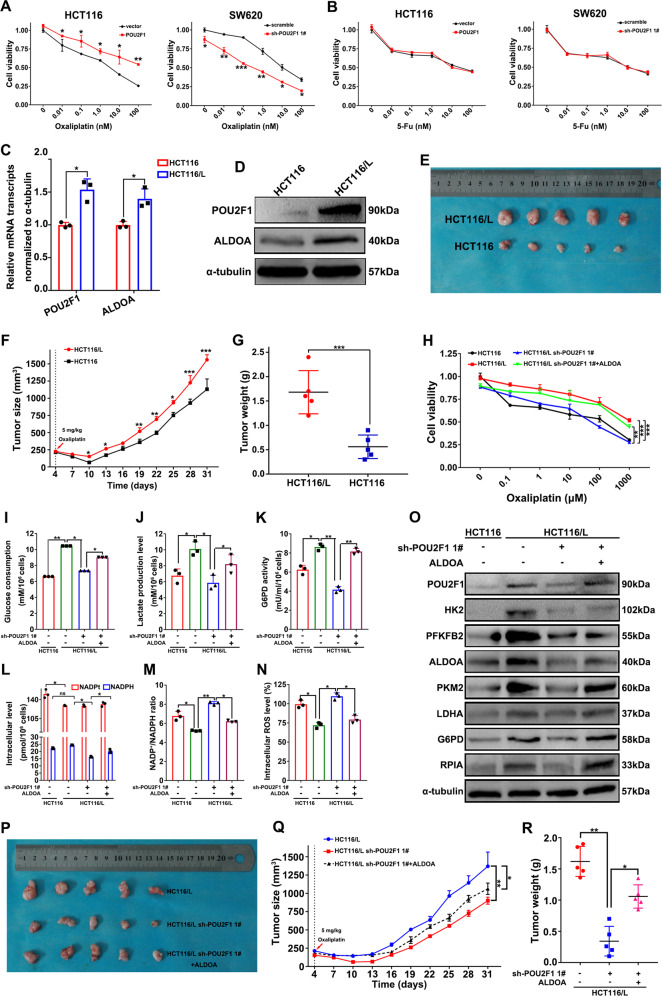
The POU2F1-ALDOA axis contributes to the oxaliplatin resistance by enhancing glycolysis and PPP activity in colon cancer. **A**, **B** POU2F1 decreased the sensitivity to oxaliplatin, but not to 5-FU in colon cancer cells, determined by CCK8. **C**, **D** RT-qPCR and Western blotting analyses of the relative levels of POU2F1 and ALDOA expression in oxaliplatin-resistant and control HCT116 cells. **E**–**G** Implantation with oxaliplatin-resistant HCT116L induced significantly bigger tumors than those with HCT116 cells. Male BALB/c nude mice were injected subcutaneously with 5 × 10^6^ cells indicated. The dynamic growth of tumors was measured longitudinally (*n* = 5 per group). At the end of observation, their tumors were dissected, photoimaged, and weighed. **H** ALDOA over-expression rescued the oxaliplatin resistance of POU2F1-silencing HCT116/L cells. **I**–**M** The levels of glucose consumption and lactate production, G6PD activity, intracellular NADPH and the ratios of NADP^+^/NADPH in the indicated cells. **N** The levels of intracellular ROS in the indicated cells. **O** Western blot analysis of the relative levels of glycolysis and PPP-related protein expression in the indicated cells. **P**–**R** ALDOA over-expression rescued the oxaliplatin resistance of POU2F1-silencing HCT116/L tumors in mice. Individual BALB/c nude mice were injected subcutaneously with 5 × 10^6^ cells indicated. When the tumors reached at 200 mm^3^, the mice were injected intraperitoneally with oxaliplatin (5 mg/kg) twice per week up to 4 weeks. The dynamic growth of tumors was measured longitudinally (*n* = 5 per group). At the end of observation, their tumors were dissected, photoimaged, and weighed. Data are representative images or expressed as the mean ± SD of each group of samples analyzed in triplicate from three separate experiments. Ns no significance, *p < 0.05, **p < 0.01, ***p < 0.001.

Antioxidant activity is closely related to chemotherapeutic resistance of cancer cells [19] and intracellular ROS levels are negatively associated with tumor chemotherapeutic resistance [20]. We hypothesized that POU2F1 could modulate the sensitivity of colon cancer cells to oxaliplatin and 5-Fu. We found that POU2F1 over-expression significantly decreased the sensitivity to oxaliplatin, but not to 5-Fu, in colon cancer cells (*p* < 0.05, Figs. 7A–B and S10A, B). Furthermore, treatment with 10 µM oxaliplatin had less cytotoxicity against POU2F1 over-expressing HCT116 cells, but had higher cytotoxicity against POU2F1-silencing SW620 cells than the controls (*p* < 0.05, *p* < 0.01, Fig. S10C). Interestingly, we detected significantly higher levels of POU2F1 and ALDOA expression in oxaliplatin-resistant HCT116/L cells than the controls (*p* < 0.05, Fig. 7C, D). As a result, implantation with HCT116/L cells induced a significantly bigger tumors than those with the same number of HCT116 cells in BALB/c nude mice (*p* < 0.05, *p* < 0.01, *p* < 0.001, Figs. 7E–G and S10D). Moreover, we explored the role of the POU2F1-ALDOA axis in oxaliplatin resistance of colon cancer cells. While oxaliplatin-resistant HCT116/L cells were less sensitive to oxaliplatin than the control HCT116 cells, POU2F1 silencing increased the sensitivity of HCT116/L cells to oxaliplatin. However, introduction of ALDOA over-expression rescued the oxaliplatin resistance in POU2F1-silencing HCT116/L cells (*p* < 0.01, *p* < 0.01, Fig. 7H). Compared with the HCT116 and HCT116/L cells, treatment with 10 µM oxaliplatin significantly decreased the viability of POU2F1-silencing HCT116/L cells at 48 and 72 h post treatments, which were partially mitigated by introduction of ALDOA over-expression (*p* < 0.01, *p* < 0.001, Fig. S10E). Thus, POU2F1 supported the oxaliplatin resistance in colon cancer cells, dependent on up-regulating ALDOA expression.

Next, we tested whether the POU2F1-ALDOA axis could modulate the glycolysis and PPP activity in oxaliplatin-resistant colon cancer. Compared with HCT116 cells, higher levels of glucose consumption and lactate production, G6PD activity and intracellular NADPH, but lower ratios of NADP^+^/NADPH and lower levels of ROS were detected in the oxaliplatin-resistant HCT116/L cells (*p* < 0.05, *p* < 0.01, Fig. 7I–N). POU2F1 silencing significantly mitigated or rescued the effects of oxalipatin resistance on glycolysis and oxidative stress in HCT116/L cells, which were blocked by introduction of ALDOA over-expression in HCT116/L cells. Western blot analysis exhibited similar patterns of the relative levels of HK2, PRKFB2, PKM2, LDHA, G6PD and RPIA expression in the different groups of cells (Fig. 7O).

To further analyze the role of the POU2F1-ALDOA axis in oxaliplatin resistance of colon cancer cells in vivo, we established xenograft colon tumors by subcutaneously implanting with HCT116/L cells, HCT116/L-sh-POU2F1 cells, and HCT116/L-sh-POU2F1 + ALDOA cells into BALB/c nude mice. Following treatment with oxaliplatin. we observed that POU2F1 silencing significantly enhanced the sensitivity of HCT116/L tumors to oxaliplatin by decreasing tumor sizes and weights (*p* < 0.05, *p* < 0.01, Figs. 7P–R and S10F), compared to HCT116/L tumors. However, the enhanced oxaliplatin sensitivity by POU2F1 silencing was partially mitigated by ALDOA over-expression in HCT116/L tumors. Hence, the POU2F1-ALDOA axis supported the oxaliplatin resistance of colon cancer cells by enhancing the glycolysis and PPP activity in vivo and in vitro.

### Both POU2F1 and ALDOA are valuable prognostic biomarkers for the survival of colon cancer patients

**Fig. 8:**
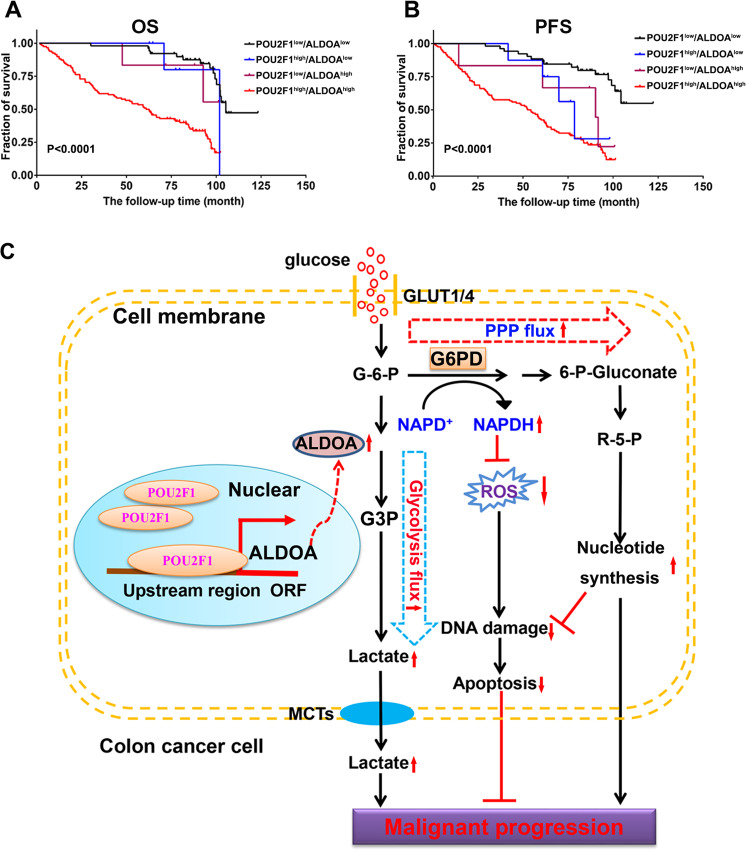
High-POU2F1 and ALDOA expression is associated with the worst prognosis of colon cancer patients. Based on the levels of POU2F1 and ALDOA expression by IHC, 184 Colon cancer patients were stratified into the POU2F1^high^/ALDOA^high^ (*n* = 118), POU2F1^high^/ALDOA^low^ (*n* = 8), POU2F1^low^/ALDOA^high^ (*n* = 6), and POU2F1^low^/ALDOA^low^ (*n* = 52). **A**, **B** Their OS and PFS were analyzed. Data are survival curves of each group of patients. **C** A schematic diagram illustrates the mechanisms by which the POU2F1-ALDOA axis regulates the malignancy and oxliplatin resistance of colon cancer. POU2F1 enhances the glycolytic flux and PPP flux by up-regulating the ALDOA expression through directly binding to its promoter region. As a result, the enhanced glycolysis enhances the production of lactate, which can promote the proliferation and induce the oxliplatin resistance in colon cancer. Meanwhile, POU2F1 enhances the PPP activity to increase the production of NADPH, which can reduce the intracellular ROS levels and cancer cell apoptosis. The enhanced PPP activity also increases the production of R-5-P to enhance DNA synthesis, ultimately contributing to the proliferation and oxliplatin resistance in colon cancer.

To understand the clinical significance of the POU2F1-ALDOA axis, we stratified 184 colon cancer patients into (1) POU2F1^high^/ALDOA^high^, (2) POU2F1^high^/ALDOA^low^, (3) POU2F1^low^/ALDOA^high^, (4) POU2F1^low^/ALDOA^low^ groups, based on the levels of POU2F1 and ALDOA expression in their colon cancer tissues. We found that the patients in the POU2F1^low^/ALDOA^low^ group had the longest OS and PFS, while the patients in the POU2F1^high^/ALDOA^high^ group had the worst outcome in this population (Fig. 8A, B). Therefore, both POU2F1 and ALDOA expression levels may be valuable biomarkers for the prognosis of colon cancer.

## Discussion

Previous studies have shown that transcription factors in the POU domain family are critical for cancer initiation and progression [21, 22]. For example, OCT4 is important for stemness of cancer stem cells [23] and POU2F1 expression is up-regulated in head and neck squamous carcinoma [24, 25]. In this study, we found that POU2F1 expression was up-regulated in colon cancer, and associated with worse prognosis. Our data support the notion that POU2F1 acts as an oncogenic factor to promote the progression of colon cancer and may be a valuable therapeutic target for colon cancer.

Metabolic reprogramming (Warburg effect) can facilitate tumor cell growth by generating more ATP, providing precursors for macromolecular synthesis and reducing ROS production in tumor cells [26, 27]. A previous study has pointed that some oncogenes increase the expression of specific glucose transporters and glycolytic enzymes to enhance glycolysis and promote cancer cell proliferation [28]. In our study, we found that POU2F1 over-expression enhanced glycolysis and PPP activity while POU2F1 silencing had opposite effects on colon cancer cells. These data were consistent with findings that oncogenic transcription factors are crucial for energy homeostasis [29, 30]. These findings also suggest that the enhanced glycolysis and PPP activity by POU2F1 may contribute to the enhanced malignant behaviors of colon cancer cells.

ALDOA is crucial for the process of glycolysis, gluconeogenesis, and the PPP [31]. We found that ALDOA expression was up-regulated in colon cancer tissues, consistent with previous observations [32, 33]. Functionally, ALDOA also promoted the proliferation, clonogenicity, glycolysis and PPP activity, suggesting that ALDOA may act as an oncogenic factor to enhance the malignant behaviors of colon cancer. Interestingly, we found that POU2F1 and ALDOA expression were paralleled in colon cancer tissues and cell lines; altered POU2F1 expression modulated ALDOA expression in colon cancer cells; both POU2F1 and ALDOA expression appeared to a better biomarker for prognosis of colon cancer. Mechanistically, POU2F1 bound the ALDOA promoter region and enhanced the ALDOA promoter activity, supporting the notion that POU2F1 can induce the transcription of several genes in cell-specific manner [34, 35]. Furthermore, POU2F1 enhanced the survival, proliferation, and clonogenicity of colon cancer cells by up-regulating ALDOA expression to enhance the glycolysis and PPP activity, and attenuating the DNA damage-induced apoptosis. Together, these data indicate that POU2F1 promotes the malignant behaviors of colon cancer by up-regulating the ALDOA expression, and the POU2F1 and ALDOA forms the axis to enhance the progression of colon cancer. To the best of our knowledge, this was the first evidence to demonstrate that POU2F1 induced ALDOA expression to promote the malignant behaviors of colon cancer cells.

Chemoresistance remains a major impediment in the treatment of many types of cancers. It is well known that oxidative stress can induce tumor cell apoptosis. Oxaliplatin is a third-generation platinum analog for treatment of advanced colon cancer, and can enhance ROS production to induce DNA damages and apoptosis in colon cancer cells. Oxaliplatin can activate apoptosis signal-regulated kinase 1 (ASK1) and c-Jun N-terminal kinase (JNK), but reduce B-cell lymphoma-2 (BcL-2) expression [36, 37]. Furthermore, previous studies have shown that the POU3F2 (BRN2), one member of the POU domain family, can increase anoikis resistance in melanoma [38] and the POU4F1 reduces the resistance to trastuzumab by activating the mitogen-activated or extracellular signal-regulated protein kinase kinases 1 and 2 (MEK1/2) and extracellular-regulated kinase 1/2 (ERK1/2) signaling in HER2-positive breast cancer [39]. In our study, we found that POU2F1 or ALDOA over-expression not only reduced intracellular ROS, but also decreased γ-H_2_AX, a hallmark of DNA damage-related apoptosis in colon cancer cells. In contrast, POU2F1 or ALDOA deficiency had opposite effects and increased the frequency of apoptotic colon cancer cells. More importantly, POU2F1 or ALDOA also enhanced the oxaliplatin resistance in colon cancer cells in vitro. Hence, the POU2F1-ALDOA axis enhanced the oxaliplatin resistance in colon cancer cells by increasing glycolysis and PPP activity. The effects of the POU2F1-ALDOA axis on glycolysis and PPP activity were mediated by up-regulating the expression of glycolysis-related enzymes (HK2, PFKFB2, PKM2, and LDHA) and PPP-related enzymes (G6PD and RPIA), and down-regulating intracellular ROS levels and oxaliplatin-induced apoptosis. Actually, the enhanced glycolysis is associated with increasing drug resistance by increasing drug efflux and DNA damage repair, drug metabolism, drug target epigenetic alterations and mutations, survival pathway activation, leading to evasion of cell death [40]. Targeting glycolysis is a new strategy for preventing drug resistance in cancer cells [41]. Conceivably, the POU2F1-ALODA axis may be promising targets for overcoming oxaliplatin resistance in treatment of colon cancer.

It is notable that an alternation in the expression of POU2F1 or ALDOA did not significantly change the sensitivity to 5-FU in colon cancer cells. The different effects of the POU2F1-ALDOA axis on chemoresistance may stem from the varying pharmacological actions between oxaliplatin and 5-FU. While oxaliplatin can induce oxidative stress and DNA damages [37], 5-FU mainly inhibits thymidylate synthase (TS) and incorporates its metabolites into RNA and DNA [42]. The POU2F1-ALDOA axis attenuated intracellular ROS production and subsequent DNA damages and apoptosis, leading to the oxaliplatin resistance in colon cancer cells. It is possible that this axis may have little effect on the 5-FU-mediated inhibition of TS. We are interested in further investigating the molecular mechanisms by which the POU2F1-ALDOA axis regulates the chemoresistance in colon cancer.

Our study provided evidence to demonstrate that POU2F1 induced ALDOA expression to form the POU2F1-ALDOA axis. The formed POU2F1-ALDOA axis promoted malignant behaviors and oxaliplatin resistance in colon cancer by enhancing glycolysis and PPP activity and protecting from oxidative stress-induced DNA damages and apoptosis (Fig. 8C). Such novel findings may provide new insights into the molecular mechanisms by which the POU2F1-ALDOA axis promotes malignant behaviors and oxaliplatin resistance of colon cancer. Therefore, our findings also may uncover new therapeutic targets for intervention of colon cancer to overcome oxaliplatin resistance.

## Materials and methods

### Data collection and analysis

Data on the POU2F1 expression in colon adenocarcinoma were obtained from The Cancer Genome Atlas (TCGA, https://gdc.cancer.gov/). POU2F1 mRNA transcripts in colon cancer and non-tumor colonic tissues were collected from the Gene Expression Omnibus datasets GSE121842, GSE5206, GSE9348, GSE20916, GSE6988 and GSE17538 (https://www.ncbi.nlm.nih.gov/geo). The DEGs in GSE121842, GSE9348 and GSE20916 were identified by a fold change of ≥1.5, or ≤−1.5 and *p* value < 0.05, determined by Student’s *t* test. The transcriptome profiles of POU2F1 silencing in SW620 cells were analyzed. Hierarchical clustering of DEGs was performed using the package R software and expressed by heatmap. Volcano maps were drawn using the ggplot2 packages. The colon cancer cases in the Molecular Signature Database were stratified into high and low-POU2F1 groups, based on the median value in the GSE121842 database. The expression profiles in gene sets named c2.kegg.v6.0.symbols.gmt between the high-POU2F1 and low-POU2F1 groups were analyzed by GSEA software (the Broad Institute, http://www.broadinstitute.org/gsea/index.jsp). The data from TCGA and GSE5206 were transformed by log_2_, and analyzed using Excel and GraphPad Prism 7.0 software. The potential functions of DEGs in POU2F1 silencing SW620 cells were analyzed by the GO and KEGG.

### Patient samples

A total of 184 surgical colon cancer tissues and 40 adjacent non-tumor tissues were collected from the Affiliated Cancer Hospital of Xiangya School of Medicine, Central South University from 2007 to 2011. Their demographic and clinical characteristics are shown in Table 1. Their colon tissues were fixed and paraffin-embedded. Those patients were followed up with a mean follow-up time of 66.38 months (0.56–106.97 months). Fresh colon cancer and matched adjacent non-tumor tissues were obtained from other 42 colon cancer patients and immediately frozen in liquid nitrogen for subsequent real-time PCR and Western blotting. All patients did not receive preoperative radiotherapy and chemotherapy. Written informed consent was obtained from individual patients. The experimental protocols were approved by the Joint Ethics Committee of the Affiliated Cancer Hospital of Xiangya School of Medicine, Central South University and Hunan Cancer Hospital in China.

### Measurements of glucose consumption, lactate production, G6PD, and intracellular NADPH levels

Following transfection, colon cancer cells (1 × 10^6^ cells/well) were incubated for 24 h. Glucose and lactate levels in the supernatants of cultured cells and its intracellular levels were measured using the Glucose Colorimetric Assay Kit (BioVision, Milpitas, USA) and Lactate Assay kit (BioVision, Milpitas, USA), respectively. Some cells were lyzed and the levels of G6PD enzymatic activity and intracellular NADPH in cell lysates were determined using G6PD Assay Kit (ab102529, Abcam, UK) and Amplite TM Colorimetric NADP/NADPH Ratio Assay Kit (ab65349, Abcam, UK), respectively.

### Intracellular ROS levels

Following transfection, some colon cancer cells (1 × 10^6^ cells/well) were cultured in 6-well plates for 48 h. Their intracellular ROS levels were quantified using CellROX Green Reagent (Life Technologies). The cells were photoimaged under a fluorescent microscope (Leica, Heidelberg, Germany) and the mean fluorescent signals in individual cells were analyzed using ImageJ software.

### Apoptosis analysis by flow cytometry

The frequency of apoptotic cells was analyzed by flow cytometry [43]. Briefly, following treatment, the cells in each group were stained with Annexin V-FITC and 7-AAD and analyzed in a Cytomics FC500 Flow Cytometry System (Beckman Coulter, Krefeld, Germany).

### Seahorse analysis

The levels of glycolysis in individual groups of cells were measured using Seahorse XF technology in a Seahorse XF-96 extracellular flux analyzer (Agilent, Santa Clara, CA, USA), according to manufacturers’ protocol. Briefly, the cells in each group were cultured in triplicate in the conditional medium and CO_2_-free condition for one h and calibrated. Following injection with glucose, oligomycin and 2-DG (Sigma), the ECAR in individual groups of cells was measured. The data were normalized against the cell densities.

### Dual-luciferase and ChIP assays

The activity of the −1900 to +50 regions of the ALDOA promoter was tested by dual-luciferase assays. Individual groups of cells were co-transfected with the plasmids for one fragment in the region to control luciferase expression, together with the Renilla luciferase reporter for 24 h. The levels of firefly and Renilla luciferase activities were measured using a Dual-Luciferase Reporter Assay System (Promega) [43].

The binding of POU2F1 to the ALDOA promoter was analyzed by ChIP using a Chromatin Immunoprecipitation Assay Kit (EMD Millipore), according to the manufacturer’s instruction. Briefly, individual groups of cells (1 × 10^7^/tube) were harvested and their genomic DNA was extracted and reacted with anti-POU2F1 or control IgG. After IP, the contained DNA was analyzed by PCR using the primers. The sequences of primers were 5′-TAGCACTTTGGGAGGCCAAG-3′ (sense) and 5′-TGGGTTCAAGCGATTCTCCC-3′ (anti-sense) for site 1#; 5′-CCTGGGAGATGAGCGAAAATC-3′ (sense) and 5’-CGAACTCCTGACCTCAAGTGAT-3’ (anti-sense) for Site 2#; 5′-TGCGAAACCCTGTCTCTACT-3′ (sense) and 5′-GCCTCCCGTGTTCAAGTGA-3′ (anti-sense) for Site 3#; 5′-AATCTCAGCTACTCGGGAGG-3′ (sense) and 5′-TTCGCTCATCTCCCAGGC-3′ (anti-sense) for Site 4#; and 5′-CGCCTGTAATCCCAGCTACT-3′ (sense) and 5′-AGTCTCGCTCTGTCGCCC-3′ (anti-sense) for Site 5#. The PCR products were resolved on 2% agarose gels [43].

### In vivo xenograft model of colon cancer and PET/CT (positron emission tomography-computed tomography) study

Male BALB/c nude mice at 4–6 weeks of age were obtained from Animal Experiment Center of Hunan Cancer Hospital and housed in a specific pathogen-free room in the animal research center of our hospital. Individual mice were injected subcutaneously with the POU2F1-silencing or scrambler siRNA-transfected SW620 cells (5 × 10^6^ cells/mice, *n* = 5/group). The mice were monitored every 4 days for the growth of implanted colon cancer up to five weeks. One day before euthanasia, individual mice were starved for 6 h and subjected to micro-PET/CT imaging for 1 h using the ^18^F-FDG probe at a dose of 6 µCi/g body weight. The data were analyzed by PET-CT software and expressed as the SUV (standardized uptake value). Subsequently, the mice were euthanized, and their tumors were dissected, photoimaged and weighed. The tumor tissues were subjected to immunohistochemistry (IHC).

In addition, male BALB/c nude mice (4–5 weeks old) were injected subcutaneously with HCT116, POU2F1-silencing HCT116/L-sh-POU2F1 or POU2F1-silencing and ALDOA over-expressing HCT116/L-sh-POU2F1-ALDOA cells (5 × 10^6^ cells/mouse, *n* = 5/group). When the tumor size reached at 200 mm^3^, the mice were injected intraperitoneally with oxaliplatin (5 mg/kg) twice per week for 4 weeks. Their tumor volumes were measured every 3 days beginning at the treatment initiation until the mice were euthanized in a blinded manner. The tumor volumes (*V*) were calculated by the formula *V* = 1/2 *L* × *W*^2^, where *W* represents the largest tumor diameter and *L* represents the second largest tumor diameter. Tumors were dissected out and weighed. The harvested tumors were immediately fixed in 10% formalin for IHC. All animal experiments were approved by the Animal Research and Care Committee of the Affiliated Cancer Hospital of Xiangya School of Medicine, Central South University and Hunan Cancer Hospital (approval number KYJJ-2018-019).

### Additional materials and method

The other material and methods are detailed in Supplementary materials. The information of primers and antibodies are listed in Tables S1 and S2.

### Statistical analysis

Data are presented as the mean ± standard deviation. The difference between groups was analyzed by Student’s *t* test. The association between the levels of POU2F1 and ALDOA expression, and the values of clinicopathological parameters were analyzed by the Chi-square test. The relationship between the levels of POU2F1 and ALDOA expression was analyzed by Spearman’s rank test. Survival was estimated by the Kaplan–Meier method and compared by log-rank test. The potential risks of individual factors for the survival of colon cancer patients were analyzed by univariate and multivariate analyses using Cox regression model after adjusting for baseline characteristics. All statistical analyses were performed using the SPSS version 22.0 (SPSS, Chicago, IL, USA). A *p* value of <0.05 was considered statistically significant.

## Supplementary information

Supplementary material

## Data Availability

The public datasets analyzed during the current study are available in the repositories listed below: • Gene Expression Omnibus GSE121842 GSE9348 GSE20916 GSE5206 GSE6988 GSE17538 • The Cancer Genome Atlas TCGA-COAD • GEPIA bioinformatic analysis POU2F1
