# Supplementary material for: The POU2F1-ALDOA axis promotes the proliferation and chemoresistance of colon cancer cells by enhancing glycolysis and the pentose phosphate pathway activity
Source: Oncogene. 2022 Jan 8;41(7):1024–39. doi: 10.1038/s41388-021-02148-y (PMC8837540; doi:10.1038/s41388-021-02148-y)
Supplement: Supplementary file 1 — Supplementary material [file 41388_2021_2148_MOESM1_ESM.docx]

**The POU2F1-ALDOA axis promotes the proliferation and chemo-resistance of colon cancer cells by enhancing glycolysis and the pentose phosphate pathway activity**

Jinguan Lin^1*^, Longzheng Xia^1*^, Linda Oyang^1^, Jiaxin Liang^1^, Shiming Tan^1^, Nayiyuan Wu^1^, Pin Yi^1,2^, Qing Pan^1, 2^, Shan Rao^1^, Yaqian Han^1^, Yanyan Tang^1^, Min Su^1^, Xia Luo^1^, Yiqing Yang^1^, Xiaohui Chen^1^, Lixia Yang^1^, Yujuan Zhou^1,3 #^, Qianjin Liao^1,3 #^

1 Hunan Key Laboratory of Cancer Metabolism, Hunan Cancer Hospital and the Affiliated Cancer Hospital of Xiangya School of Medicine, Central South University, Changsha, 410013, Hunan, China.

2 University of South China, Hengyang, 421001, Hunan, China.

3 Hunan Key Laboratory of Translational Radiation Oncology, 283 Tongzipo Road, Changsha 410013, Hunan, China

* These authors contribute equally to the work

#Correspondence to: Qianjin Liao or Yujuan Zhou, Hunan Key Laboratory of Cancer Metabolism, Hunan Cancer Hospital and the Affiliated Cancer Hospital of Xiangya School of Medicine, Central South University, Changsha, 410013, Hunan, China., 283 Tongzipo Road, Changsha 410013, Hunan, China. Tel: 86-731-88651681; Fax: 86-731-88651999; Email: march-on@126.com. or yujany_zhou@163.com.

This content includes:

1. Supplementary Materials and Methods
2. Ten supplementary figures
3. Two supplementary tables

**Supplementary Materials and Methods**

**Cell lines and culture**

Human colon cancer HCT116, SW620, SW480, HT-29 and LoVo cell lines were obtained from the Cancer Research Institute of Central South University and HCT116/L cells were provided by Shanghai Jiaotong University (Shanghai, China), respectively. These cell lines were identified by short tandem repeat STR test. The SW620, SW480, HCT116/L and HT-29 cells were cultured in RPMI-1640 (Gibco, USA). The HCT116, HCoEpiC, 293T and LoVo cells were cultured in DMEM (Gibco). All cell lines were cultured in medium supplemented with 12% FBS (Zeta Life, France), 100 Units/ml of penicillin, and 100 μg/ml of streptomycin (Gibco) at 37 °C in 5% CO_2_.

**Plasmids and transfection**

The full-length POU2F1 and ALDOA cDNAs were cloned into pCDH-CMV-MCS-EF1-Puro vector to generate pCDH-POU2F1 and pCDH-ALDOA plasmids, respectively, followed by DNA sequencing. The DNA fragments for the expression of POU2F1 and ALDOA-specific short hairpin RNA (shRNA) were synthesized and cloned into the plent-u6-puro vector to generate pPlent-u6-shPOU2F1 or pPlent-u6-shALDOA plasmids. The targeting sequences specific for POU2F1 were 5'-GATCCGCAACTGGGAACCTGGTATTTTCAAGAGAAATACCAGGTTCCCAGTTGCTTTTTTA-3', and the targeting sequences for ALDOA were 5'-GATCCGCGTCTCTAACCACGCCTATTATTCAAGAGATAATAGGCGTGGTTAGAGACGTTTTTTA-3'. Colon cancer cells were transfected with the control or POU2F1 or ALDOA-expressing plasmid using lipofectamine 2000 (Invitrogen, Waltham, MA, USA) and treated with 5 µg/ml of puromycin (Sigma, Saint Louis, Missouri, USA) to generate stable POU2F1 or ALDOA over-expressing cells. A similar protocol was sued to generate POU2F1 and ALDOA silencing cells.

Some POU2F1 over-expressing HCT116 cells were transfected with the control or pPlent-u6-shALDOA for 48 h to determine whether ALDOA silencing could mitigate the effect of POU2F1 over-expression on colon cancer cells. Similarly, some POU2F1 silencing SW620 cells were transfected with the control or pCDH-ALDOA plasmid for 48 h to determine whether ALDOA over-expression could rescue the function of POU2F1-silencing in colon cancer cells. Some cells were treated with H_2_O_2_ (Sigma) in saline, 5-Fu (5-fluorouracil) in saline, Oxaliplatin in 5% glucose solution (Hengrui Medicine, Jiangsu, China).

**RNA extraction and quantitative real-time PCR (RT-qPCR)**

Total cellular RNA was extracted using TRIzol reagent (Invitrogen, 15596-018), and reversely transcribed into cDNA using Revert Aid First Strand cDNA Synthesis Kit (Thermo scientific, Massachusetts, USA), according to the manufacturer’s instruction. The relative levels of targeted gene mRNA transcripts to the α-tubulin were determined by RT-qPCR using a Fast Start Essential DNA Green Master kit (Lifescience, Roche, Mannheim, Germany) and specific primers (**Table S1**) in the Roche Light Cycler® 96 Instrument (Lifescience). The PCR reactions were performed in triplicate at 95°C for 5 min and subjected to 40 cycles of 95°C (30 s), 60°C (10 s), and 72°C (1 min). The data were analyzed by 2^−∆∆Ct^.

**Western blot analysis**

The relative levels of targeting proteins were determined by Western blot assays. Individual cell lysates (30 μg) were separated by sodium dodecyl sulfate-polyacrylamide gel electrophoresis (SDS-PAGE) on 10% gels, and transferred onto polyvinylidene difluoride (PVDF) membranes. After being blocked with 5% fat-free dry milk in TBST, the membranes were incubated overnight at 4°C with primary antibodies (**Tables S2**). The bound antibodies were detected by horseradish peroxidase (HRP)-conjugated second antibodies, and visualized using Pierce™ ECL Western Blotting Substrate (Thermo Scientific). The levels of targeting proteins were quantified by densitometric scanning using ImageJ software.

**Cell viability and colony formation assays**

The proliferation of each group of colon cancer cells was examined by CCK-8 assays. Briefly, each group of cells (5000 cells/well) were cultured in 96-well plates and treated in triplicate with the indicated drugs for 48 h. After reaction with CCK-8 reagents, the cell viability in each group was measured for the absorbance at 450 nm in a microplate reader. To determine clonogenicity of individual groups of cells, the cells (2000 cells/dish) were cultured in 6-cm dishes for 10-14 days, fixed with methanol and stained with 0.1% crystal violet. The visible colonies were counted in a blinded manner.

**Immunohistochemistry (IHC)**

Individual paraffin-embedded tissue sections (4 µm) were dewaxed and rehydrated. The tissue sections were stained with primary antibodies overnight at 4 °C and the bound antibodies were detected with HRP-conjugated second antibodies, followed by visualizing with DAB (3,3' Diaminobenzidine), according to the immunohistochemical kit protocols (Cwbiotech, Beijing, China). The primary antibodies included anti-POU2F1 (1:50), anti-ALDOA (1:50), anti-HK2 (1:100), anti-LDHA (1:100) and anti-G6PD (1:50). The samples were scored by two pathologists in a blinded manner, according to the staining intensity and percent of positive cells, as described previously (43, 44). (1) staining intensity: 0, no observed cell staining; 1, cells with weak staining: 2, cells with moderate staining; 3, cells with strong staining; (2) percent of positive cells: 0, no positive cells; 1, less than 25% of positive cells; 2, between 25% and 50% positive cells; 3, positive cells over 50%. Next, the IHC score of individual sections was obtained by multiplying the intensity score and positive cell score, leading to a maximum score of 9. A section with IHC score of < 4 was designated as low expressions. A total of three sections from individual specimens were analyzed.

**Immunofluorescent assay**

Following transfection, the levels of POU2F1, ALDOA, PCNA and γ-H_2_AX in individual groups of cells were analyzed by immunofluorescent assays using antibodies against POU2F1 (1:100), ALDOA (1:50), PCNA (1:100) and γ-H_2_AX (1:100) and fluorescent-second antibodies (1:1000).

**Supplementary Figures**


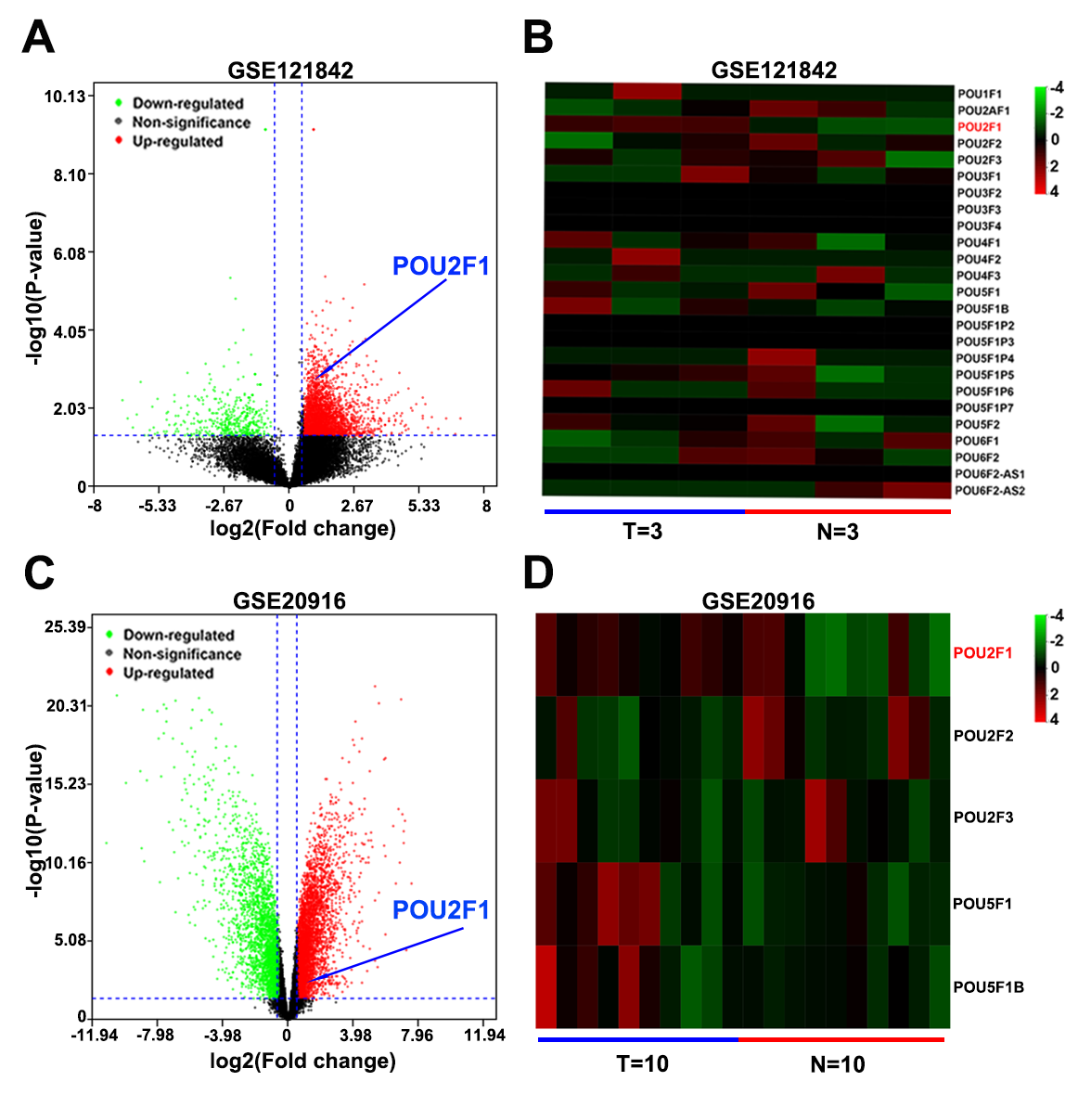


**Figure S1. Up-regulated POU2F1 expression is associated with worse prognosis of colon cancer.**

(**A**) Volcano plot illustrated the distribution of DEGs between 3 colon cancer and 3 matched non-tumor colon tissues in the GSE121842 dataset. (**B**) Heatmap analysis of the distribution of DEGs in the POU family. (**C**) Volcano plot exhibited the distribution of DEGs between 10 colon cancer and 10 matched non-tumor colon tissues in the GSE20916 dataset. (**D**) Heatmap analysis of the distribution of DEGs in the POU family between 10 colon cancer and 10 non-tumor colon tissues. The DEGs were defined, based on a fold change ≥1.5 and P <0.05.


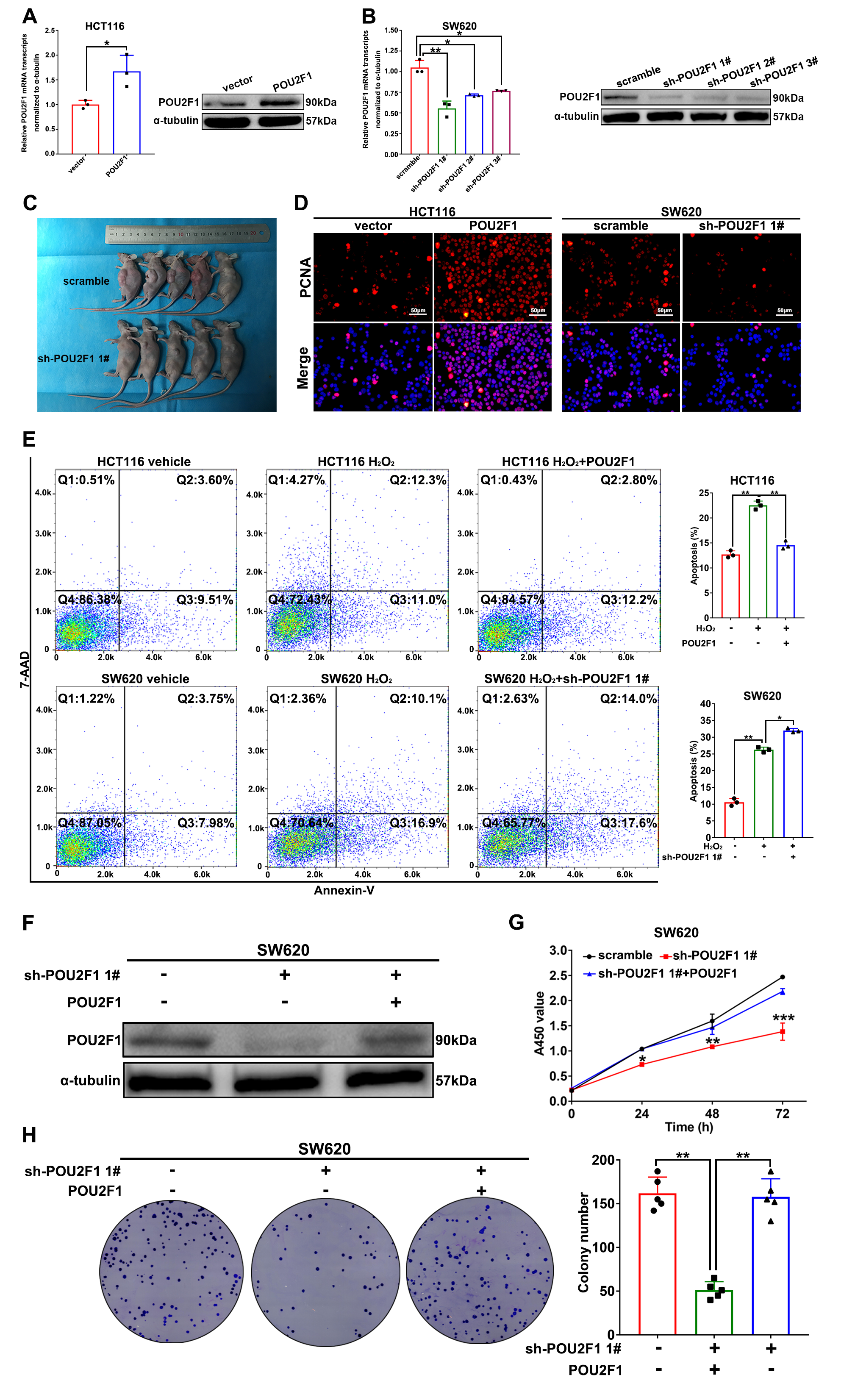


**Figure S2. Enforced POU2F1 expression enhances the proliferation of POU2F1-silencing colon cancer cells.**

(**A**) The relative levels of mRNA transcripts and protein expression verified POU2F1 over-expression in HCT116 cells. (**B**) Validation of POU2F1 silencing in SW620 cells by RT-qPCR and Western blot assays. (**C**) Photoimages of mice bearing SW620/scramble, or SW620/sh-POU2F1 1# tumors. (**D**) Immunofluorescent analysis of PCNA expression in the indicated cells (magnification x 200, scale bars 50 μm). (**E**) Flow cytometry analysis of apoptotic cells in the indicated cells following treatment with 2 mM H_2_O_2_ for 24 h. (**F**) Western blot analysis of the relative levels of POU2F1 expression in the indicated groups of SW620 cells. (**G-H**) Enforced POU2F1 expression rescued the proliferation and clonogenicity of POU2F1-silencing SW620 cells. Data are representative images or expressed as the mean ± SD of each group of samples analyzed in triplicate from three separate experiments. *P<0.05, **P<0.01, ***P<0.001.


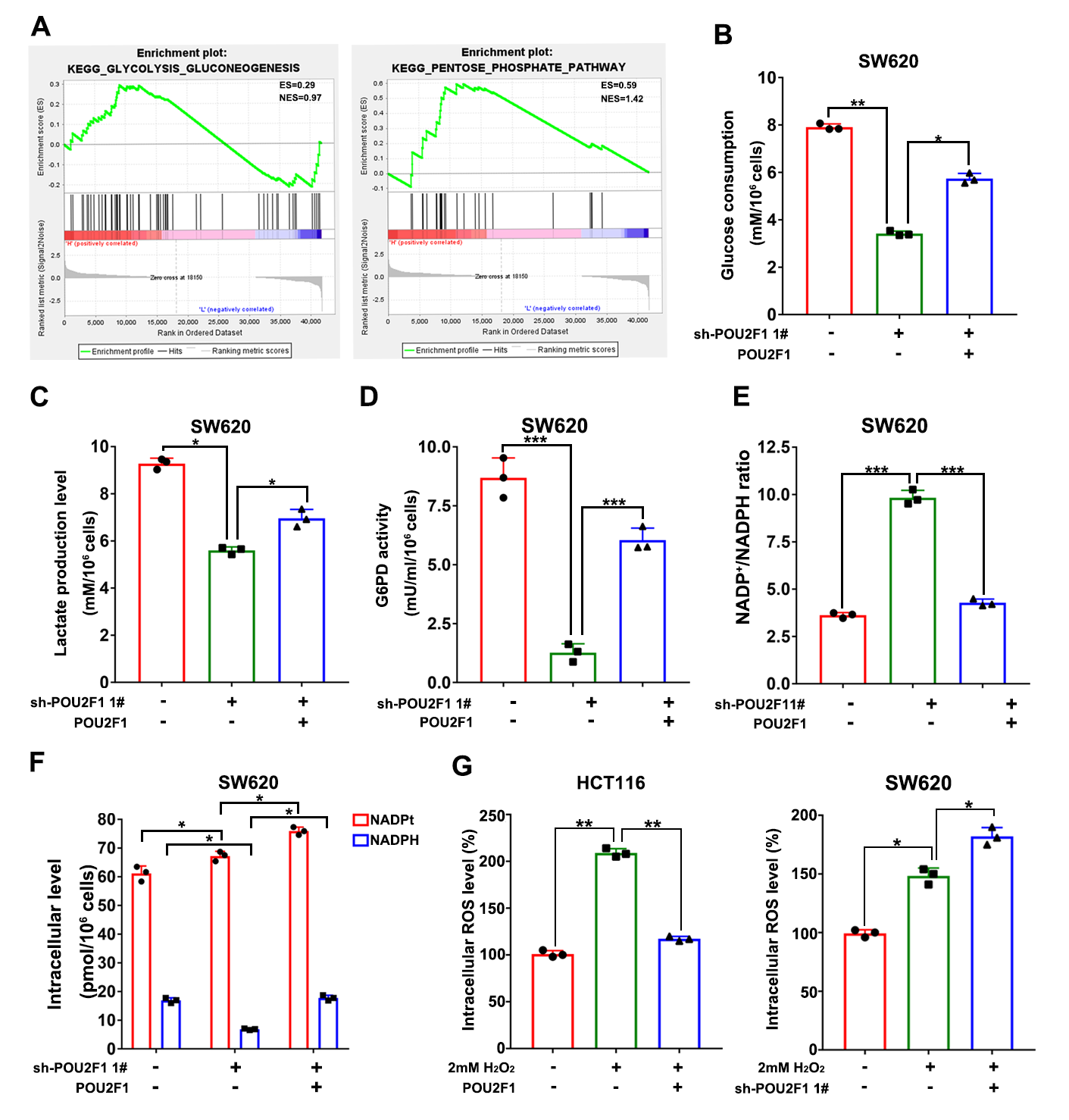


**Figure S3. Enforced POU2F1 expression rescues glycolysis and PPP activity in POU2F1-silencing colon cancer cells.**

(**A**) GSEA analysis of enriched glycolysis- and PPP-related genes in high POU2F1 expressing colon cancer of the GSE121842 dataset. (**B-F**) Enforced POU2F1 expression partially rescued the levels of extracellular glucose consumption, extracellular lactate level, G6PD activity, intracellular NADP^+^ and NADPH and reduced the ratios of NADP^+^/NADPH in POU2F1-silencing SW620 cells. (**G**) POU2F1 over-expressing HCT116 and POU2F1 silencing SW620 cells as well as their control cells were treated in triplicate with, or without, 2 mM H_2_O_2_ for 24 h. The levels of intracellular ROS were measured. The levels of ROS in the control cells without H_2_O_2_ treatment were designated as 100%. Data are representative images or expressed as the mean ± SD of each group of samples analyzed in triplicate from three separate experiments. *P<0.05, **P<0.01, ***P<0.001


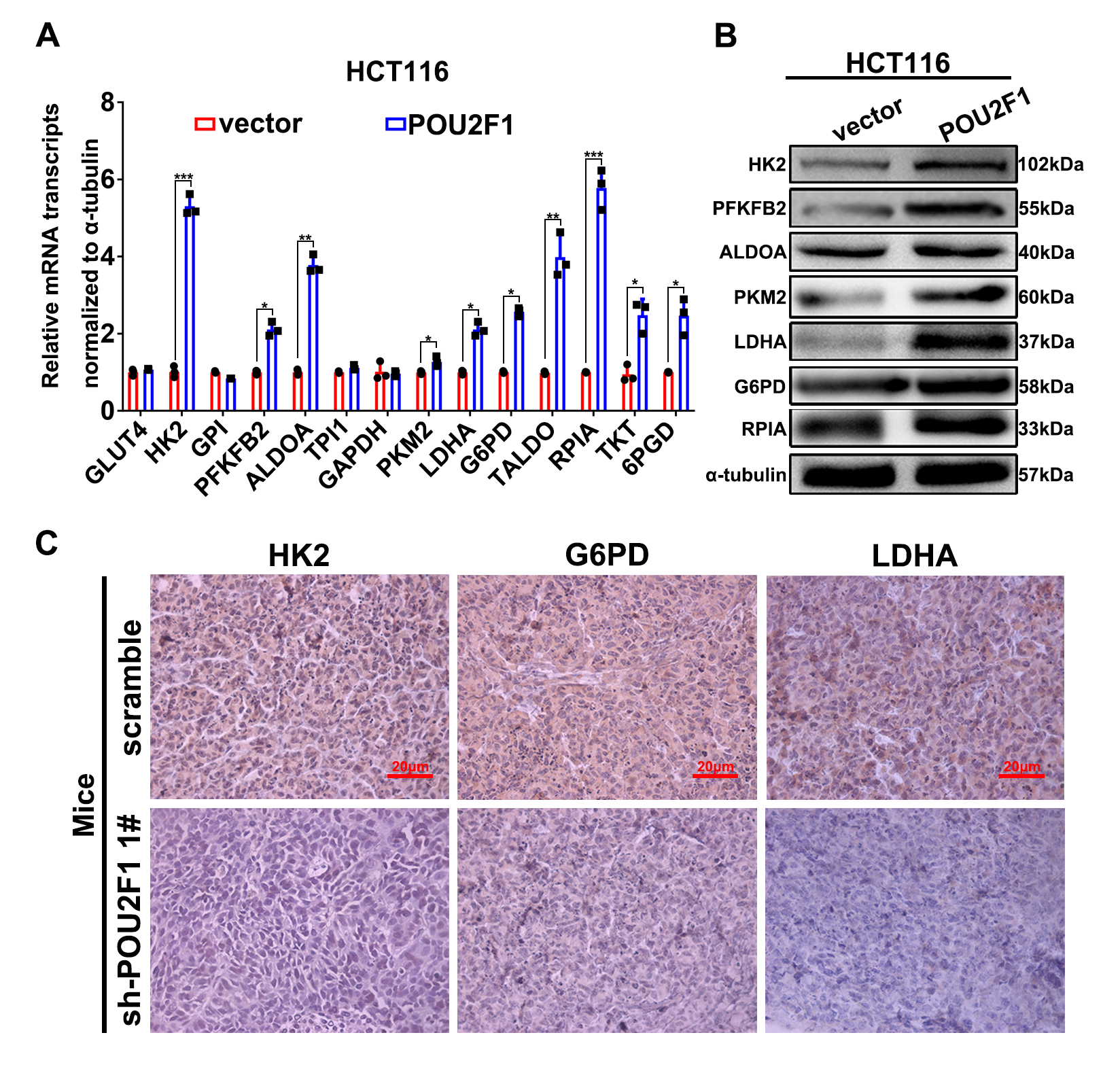


**Figure S4. The levels of ALDOA expression are paralleled with POU2F1 in colon cancer cells.**

(**A-B**) RT-qPCR and Western blot analyses of the relative levels of glycolysis- and PPP-related genes in POU2F1 over-expressing and control HCT116 cells. (**C**) IHC analysis of HK2, G6PD and LDHA in SW620 xenograft tumors (magnification x 400, scale bars 20 μm). Data are representative images or expressed as the mean ± SD of each group of cells analyzed in triplicate from three separate experiments. *P<0.05, **P<0.01, ***P<0.001.


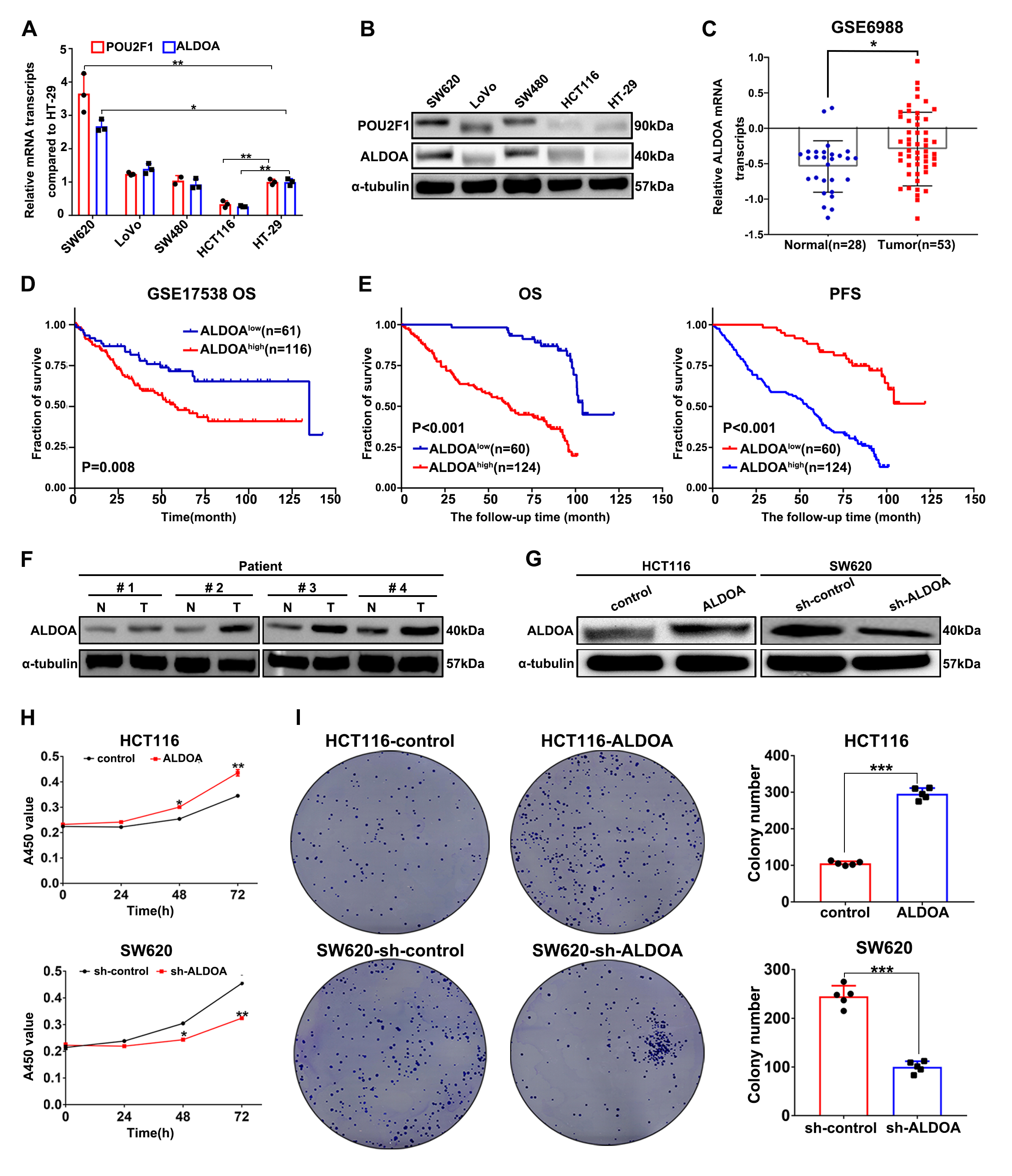


**Figure S5. Up-regulated ALODA expression is associated with worse prognosis of colon cancer.** (**A-B**) RT-qPCR and Western blotting analyses indicated the relative levels of POU2F1 expression were paralleled with ALDOA in the indicated cells. (**C**) Scatter plot exhibited the levels of ALDOA mRNA transcripts in 53 colon cancer (Tumors) and 28 non-tumor colon tissues (Normal) in GSE6988 dataset. (**D**) Kaplan-Meier analysis of OS in colon cancer patients after stratification with the median value of ALDOA mRNA transcripts in GSE17538 dataset. (**E**) High levels of ALDOA expression were associated with a shorter PFS and OS of 184 colon cancer patients. (**F**) Western blot analysis of the relative levels of ALDOA expression in colon cancer (T) and adjacent non-tumor tissues (N). (**G**) Western blot validated ALDOA over-expression in HCT116 and ALDOA silencing in SW620 cells following transfection. (**H-I**) ALDOA promoted the proliferation and colony formation of colon cancer cells, determined by CCK-8 and colony formation assays, respectively. Data are representative images or expressed as the mean ± SD of each group of samples analyzed in triplicate from three separate experiments. *P<0.05, **P<0.01, ***P<0.001.


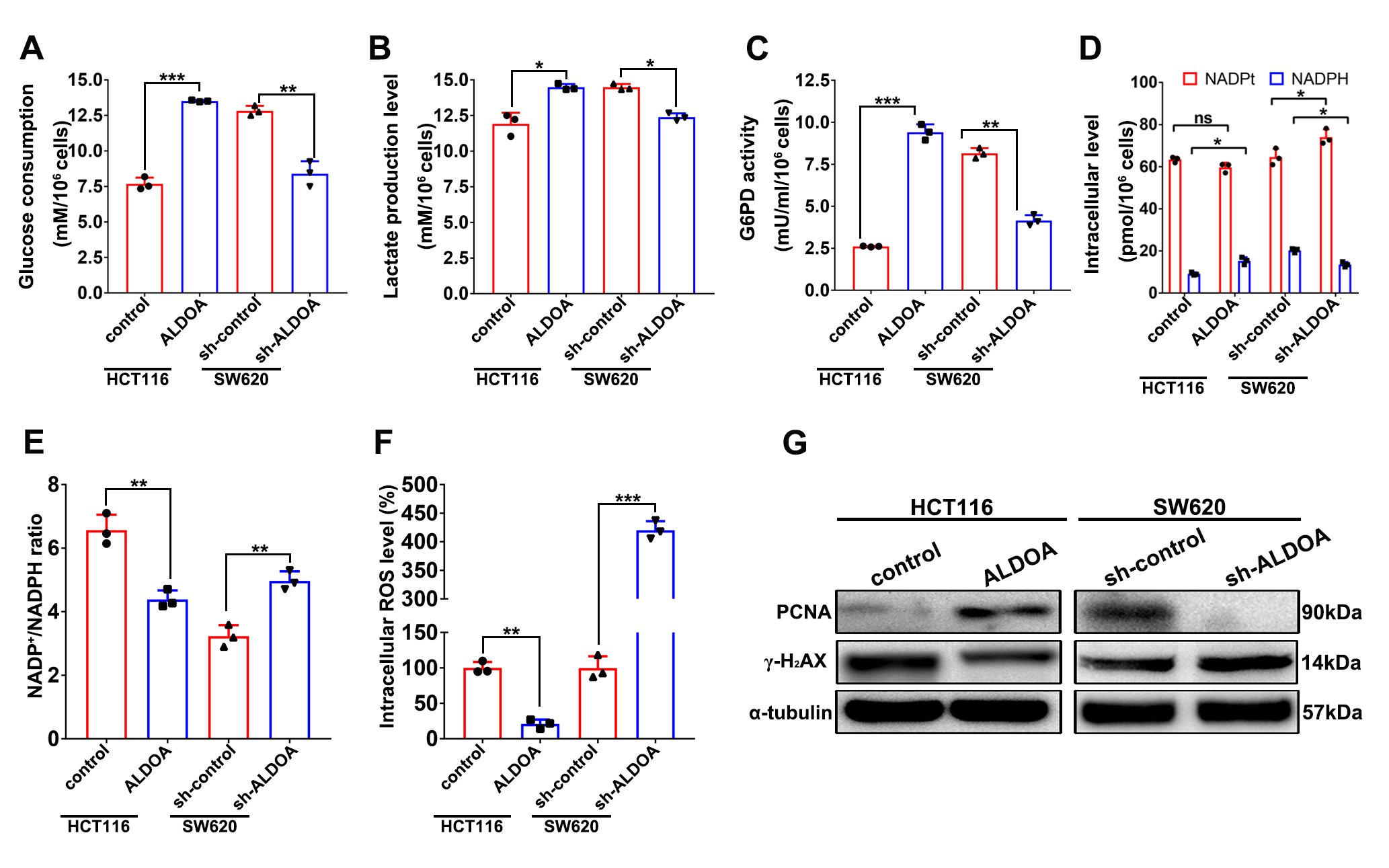


**Figure S6. Up-regulated ALODA expression** **enhances glycolysis and PPP activity in colon cancer cells.**

(**A-C**) The levels of extracellular glucose consumption, lactate and G6PD activity in the indicated cells. (**D&E**) The levels of intracellular NADP^+^ and NADPH and the ratios of NADP^+^/NADPH in the indicated cells. (**F**) Flow cytometry analysis of the levels of intracellular ROS. (**G**) Western blot analysis of the relative levels of γ-H_2_AX and PCNA expression in the indicated cells. Data are representative images or expressed as the mean ± SD of each group of samples analyzed in triplicate from three separate experiments. *P<0.05, **P<0.01, ***P<0.001.


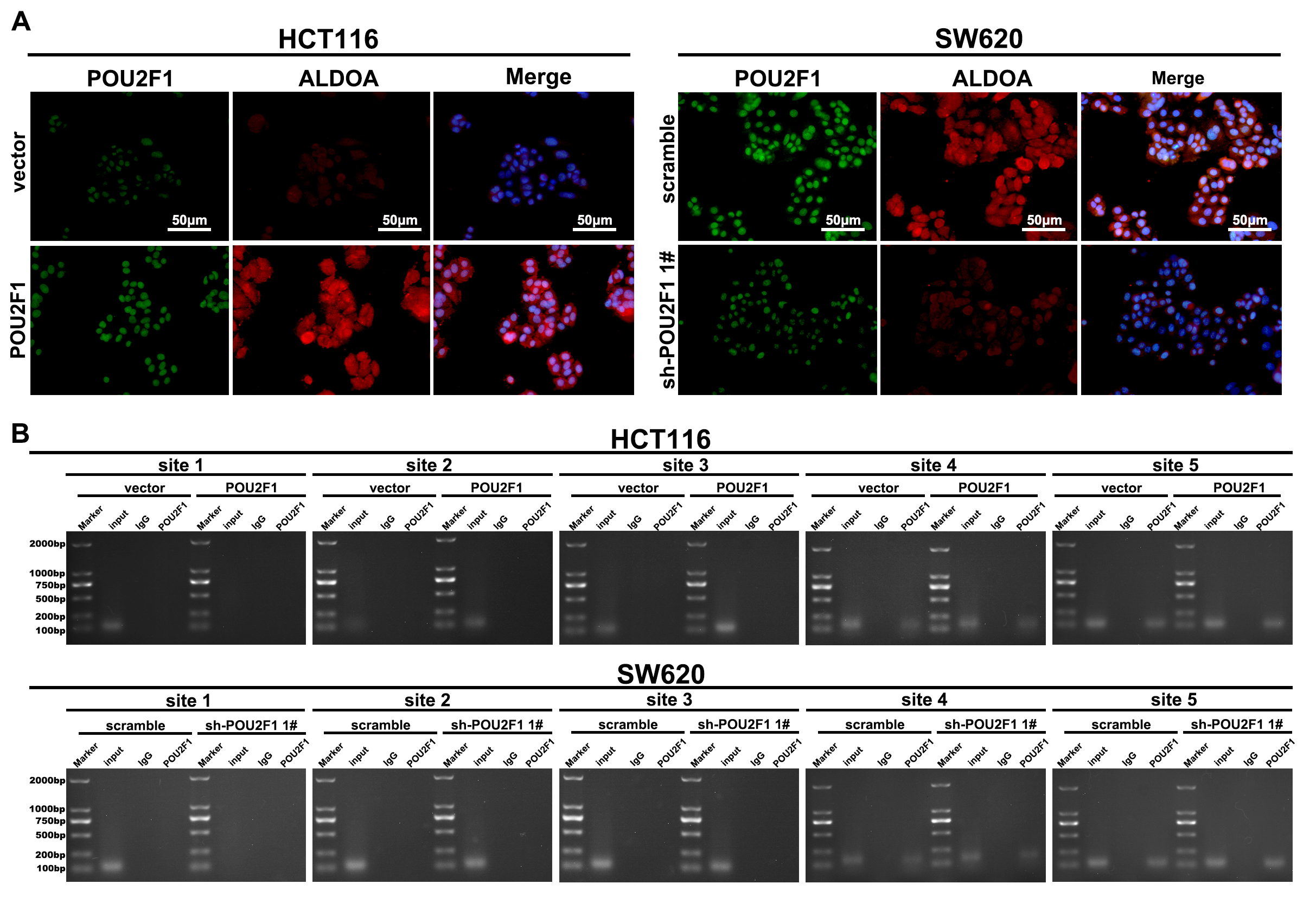


**Figure S7. POU2F1 promotes ALDOA expression in colon cancer cells.**

(**A**) Immunofluorescent analysis of POU2F1 and ALDOA expression in the indicated cells (magnification x 200, scale bars 50 μm). (**B**) Chromatin immunoprecipitation assay revealed that POU2F1 bound directly to the ALDOA promoter at sites 4, and 5 in colon cancer cells.


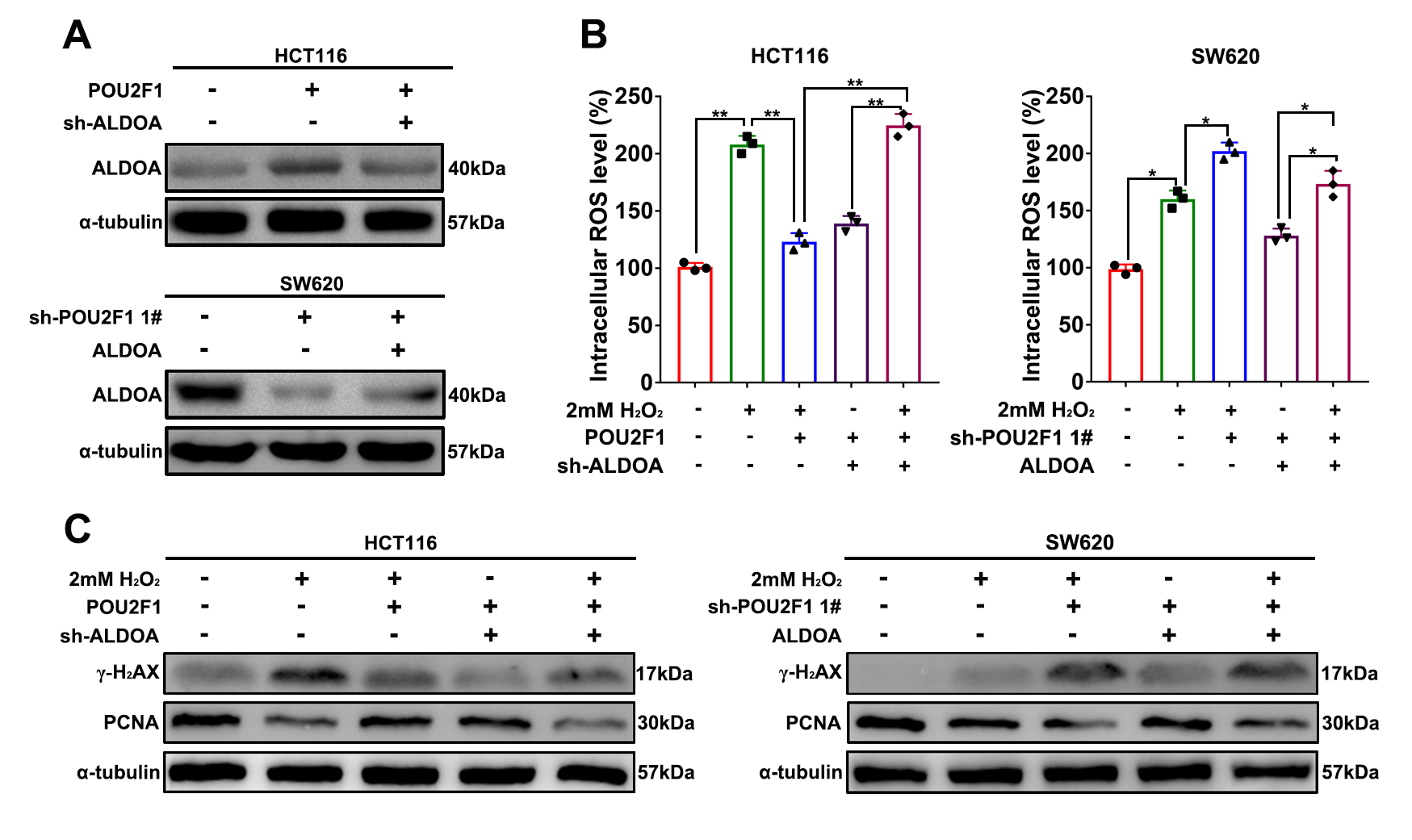


**Figure S8.** **Alternation in the levels of ALDOA expression modulates the effect of POU2F1 on PCNA and γ-H_2_AX expression in colon cancer cells.**

(**A**) Western blot analysis of the relative levels of ALDOA expression in the indicated cells. (**B**) The levels of intracellular ROS in the indicated cells following treatment with, or without, 2 mM H_2_O_2_ for 24 h. (**C**) The relative levels of PCNA and γ-H_2_AX expression in the indicated cells following treatment with, or without, 2 mM H_2_O_2_ for 24 h. Data are representative images or expressed as the mean ± SD of each group of cell samples analyzed in triplicate from three separate experiments. *P<0.05, **P<0.01, ***P<0.001.


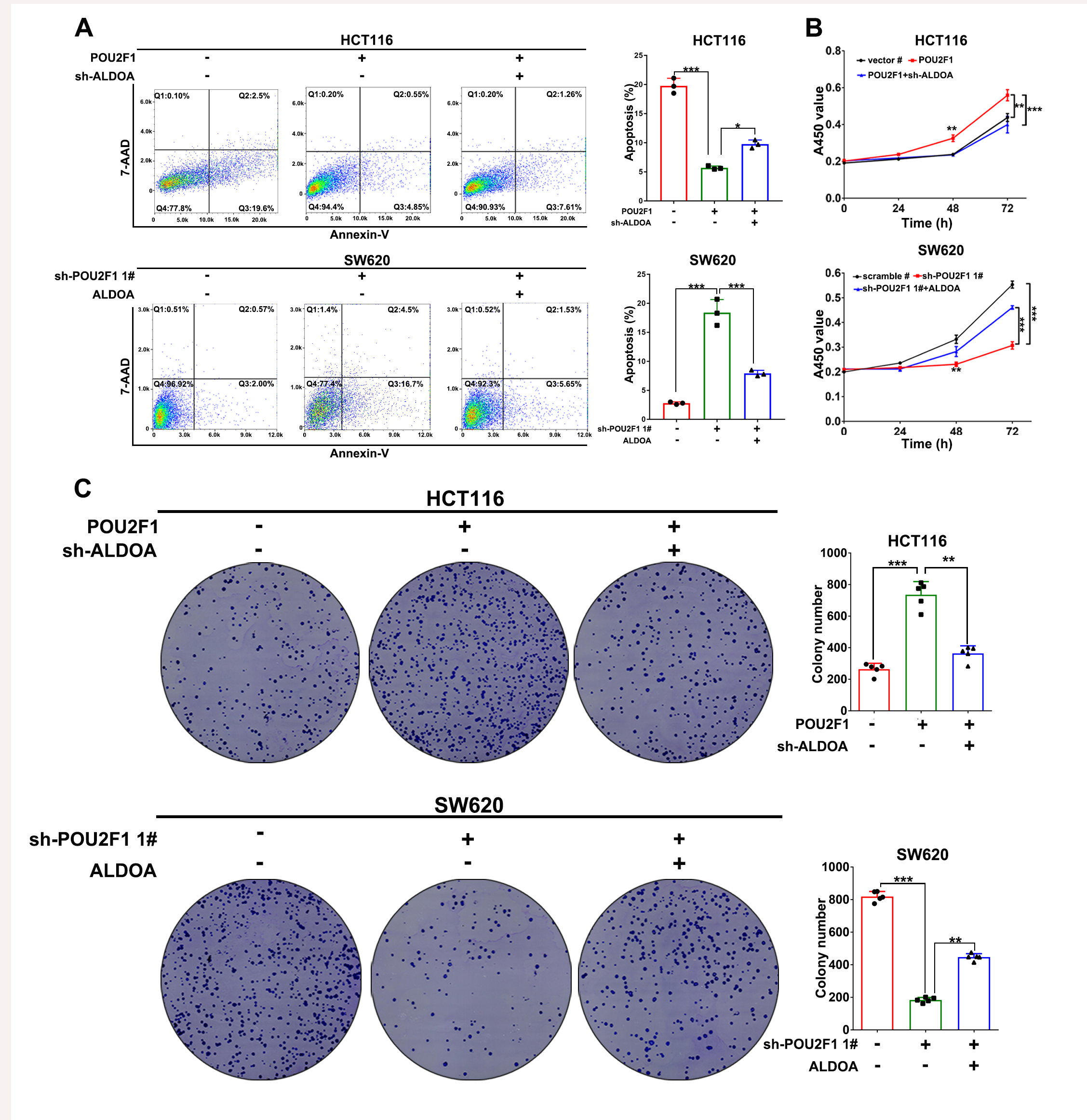


**Figure S9.** **Alternation in the levels of ALDOA expression partially reduces the effect of POU2F1 on the apoptosis, proliferation and clonogenicity of colon cancer cells.**

(**A**) Flow cytometry analysis of apoptosis in the indicated cells. (**B-C**) ALDOA silencing partially rescued apoptosis, but reduced proliferation and clonogenicity in POU2F1 over-expressing HCT116 cells while enforced ALDOA over-expression partially decreased apoptosis, but increased the proliferation and clonogenicity of POU2F1-silencing SW620 cells. Data are representative images or expressed as the mean ± SD of each group of cell samples analyzed in triplicate from three separate experiments. **P<0.01, ***P<0.001.


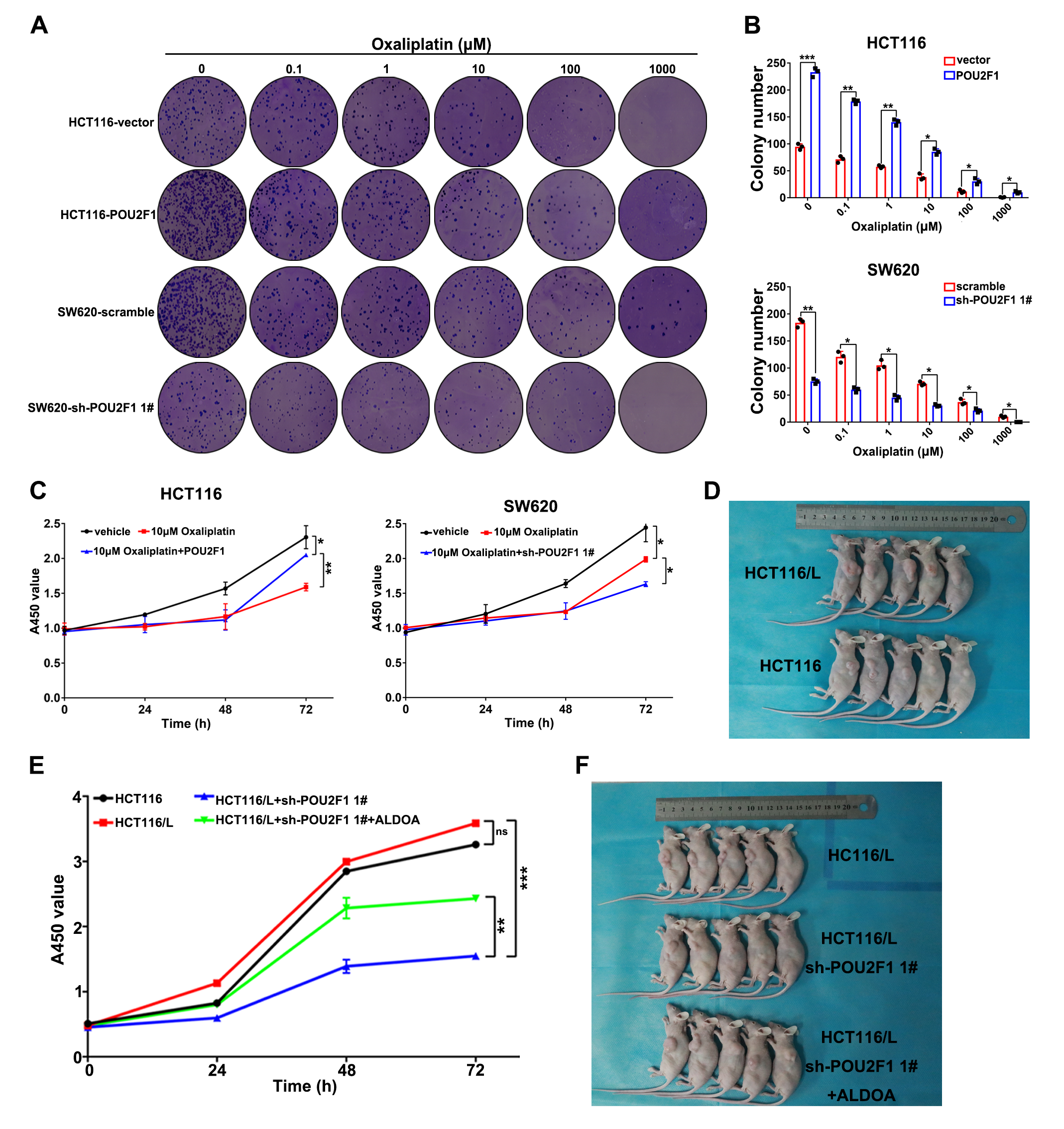


**Figure S10. The POU2F1-ALDOA axis contributes to the oxaliplatin resistance in colon cancer**

(**A-B**) POU2F1 decreased the oxaliplatin sensitivity of colon cancer cells, determined by colony formation assays. (**C**) POU2F1 modulated the sensitivity to 10 μM oxaliplatin in the indicated cells at 72 h post treatment. (**D**) Photoimages of mice bearing HCT116, or HCT116/L tumors. (**E**) ALDOA over-expression partially enhanced the proliferation of POU2F1-silencing HC T116/L cells. (**F**) Photoimages of mice bearing HCT116/L, HCT116/L-sh-POU2F1 or HCT116/L-sh-POU2F1+ALDOA tumors, following oxaliplatin treatment. Data are representative images or expressed as the mean ± SD of each group of samples analyzed in triplicate from three separate experiments. Ns: no significance, *P<0.05, **P<0.01, ***P<0.001.

Supplementary Tables

**Table S1. The sequences of primers for RT-qPCR**

| **Genes** | **Refseq No.** | **Forward (5'-3')** | **Reverse (5'-3')** |
| --- | --- | --- | --- |
| POU2F1 | NM_001198783.2 | GATCTGCCTGGTGAATGCTG | ATCGCCGCAAAACATCTCTC |
| ALDOA | NM_001243177.4 | GCAACTTTCCTCTGCCTAGC | AAGCAGAGACAGTTGAGGCT |
| GLUT4 | NM_012751.1 | CCTTGCCTCGCTGCTGTA | CCTCCTGCCTTAGTTGGTCA |
| HK2 | NM_001371525.1 | GACCAACTTCCGTGTGCTTT | TCCATGAAGTTAGCCAGGCA |
| GPI | NM_001329911.2 | TGACTGAAGCCCTTAAGCCA | ATTCGTGATGGTCTCCTGGG |
| PFKFB-2 | NM_001018053.2 | AACACGCTACCTCAACTGGA | CCTTAACATCTTCCAGCGCC |
| TPI1 | NM_000365.6 | GGGGCTTTTACTGGGGAGAT | CCAATGCAGGCGATTACTCC |
| GAPDH | NM_001037190.1 | AAGAAGGTGGTGAAGCAGGCATC | CGAAGGTGGAAGAGTGGGAGTTG |
| PKM2 | NM_001206796.2 | ATGGCTGACACATTCCTGGA | AGAAGTTCAGACGAGCCACA |
| LDHA | NM_001165416.1 | CAACATGGCAGCCTTTTCCT | ACCCACCCATGACAGCTTAA |
| G6PD | NM_001042351.2 | TCAACAGCCACATGAATGCC | ACAGGGAGGAGATGTGGTTG |
| TALDO | NM_006755.1 | AGCTGTCATCAACCTGGGAA | GCCACATGCCAATCAAGGAT |
| RPIA | NM_144563.2 | ATCCCAATGGCCTATGTCCC | TCCATTTGTGTACCCGGTCA |
| TKT | NM_001064.3 | TCCGAGAGCAACATCAACCT | CTCTGTAGCAACGCCATCAC |
| 6PGD | NM_002631.3 | CCGGAACTTCAGAACCTCCT | TGTGGATAAACTGCCCTGGT |
| α-tubulin | NM_006009.4 | TACGGAAAGAAGTCCAAGC | CTGAGGGAAGCAGTGATG |

**Table S2. Antibodies for Western blotting, chromatin immunoprecipitation, immunofluorescence, and immunohistochemistry**

| **Antibody** | **Catalog number** | **Company** | **Experiment** | **Dilution** |
| --- | --- | --- | --- | --- |
| anti-POU2F1 | #8157 | CST | Western Blotting | 1:500 |
|  |  |  | ChIP assay | 30 μg/ml cell lysate |
|  |  |  | Immunofluorescence | 1:100 |
|  | ab178869 | Abcam | Immunohistochemistry | 1:50 |
| anti-ALDOA | #3188 | CST | Western Blotting | 1:500 |
|  |  |  | Immunofluorescence | 1:50 |
|  | ab273636 | Abcam | Immunohistochemistry | 1:50 |
| anti-HK2 | ab209847 | Abcam | Western Blotting | 1:500 |
|  |  |  | Immunohistochemistry | 1:100 |
| anti-PFKFB2 | #13029 | CST | Western Blotting | 1:500 |
| anti-PKM2 | #4053 | CST | Western Blotting | 1:500 |
| anti-LDHA | #3582 | CST | Western Blotting | 1:500 |
|  |  |  | Immunohistochemistry | 1:100 |
| anti-G6PD | ab133525 | Abcam | Western Blotting | 1:500 |
|  |  |  | Immunohistochemistry | 1:50 |
| anti-RPIA | 13010-1-AP | Proteintech | Western Blotting | 1:400 |
| anti-PCNA | #13110 | CST | Western Blotting | 1:500 |
|  |  |  | Immunofluorescence | 1:100 |
| anti-γ-H_2_AX | ab11175 | Abcam | Western Blotting | 1:500 |
|  |  |  | Immunofluorescence | 1:100 |
| anti-α-tubulin | AF0001 | Beyotime Biotechnology | Western Blotting | 1:1000 |
| HRP-labeled Goat Anti-Rabbit IgG(H+L) | A0208 | Beyotime Biotechnology | Western Blotting | 1/5000 |
| HRP-labeled Goat Anti-Mouse IgG(H+L) | A0216 | Beyotime Biotechnology | Western Blotting | 1/5000 |
| Goat anti-Rabbit IgG (H+L) Alexa Fluor 488 | A32731 | Invitrogen | Immunofluorescence | 1/1000 |
| Goat anti-Rabbit IgG (H+L) Alexa Fluor 594 | A11012 | Invitrogen | Immunofluorescence | 1/1000 |
